# Financial risk protection in health care in Bangladesh in the era of Universal Health Coverage

**DOI:** 10.1371/journal.pone.0269113

**Published:** 2022-06-24

**Authors:** Taslima Rahman, Dominic Gasbarro, Khurshid Alam

**Affiliations:** 1 Murdoch Business School, Murdoch University, Perth, Western Australia, Australia; 2 Institute of Health Economics, University of Dhaka, Dhaka, Bangladesh; Imam Abdulrahman Bin Faisal University, SAUDI ARABIA

## Abstract

**Background:**

Ensuring financial risk protection in health care and achieving universal health coverage (UHC) by 2030 is one of the crucial Sustainable Development Goals (SDGs) targets for many low- and middle-income countries (LMICs), including Bangladesh. We examined the critical trajectory of financial risk protection against out-of-pocket (OOP) health expenditure in Bangladesh.

**Methods:**

Using Bangladesh Household Income and Expenditure Survey data from 2005, 2010, and 2016, we examined the levels and distributions of catastrophic health expenditure (CHE) and impoverishment incidences. We used the normative food, housing, and utilities method, refining it by categorizing households with zero OOP expenses by reasons.

**Results:**

OOP expenditure doubled between 2005 and 2016 (USD 115.6 in 2005, USD 162.1 in 2010, USD 242.9 in 2016), accompanied by rising CHE (11.5% in 2005, 11.9% in 2010, 16.6% in 2016) and impoverishment incidence (1.5% in 2005, 1.6% in 2010, 2.3% in 2016). While further impoverishment of the poor households due to OOP expenditure (3.6% in 2005, 4.1% in 2010, 3.9% in 2016) was a more severe problem than impoverishment of the non-poor, around 5.5% of non-poor households were always at risk of impoverishment. The poorest households were the least financially protected throughout the study period (lowest vs. highest quintile CHE: 29.5% vs. 7.6%, 33.2% vs. 7.2%, and 37.6% vs. 13.0% in 2005, 2010, and 2016, respectively). The disparity in CHE among households with and without chronic illness was also remarkable in 2016 (25.0% vs. 9.1%).

**Conclusion:**

Financial risk protection in Bangladesh exhibits a deteriorated trajectory from 2005 to 2016, posing a significant challenge to achieving UHC and, thus, the SDGs by 2030. The poorest and chronically ill households disproportionately lacked financial protection. Reversing the worsening trends of CHE and impoverishment and addressing the inequities in their distributions calls for implementing UHC and thus providing financial protection against illness.

## Introduction

Protecting people from the financial risks of seeking health care is a crucial health system goal [1]. As an ethical and social imperative, financial risk protection in health care is also a critical component of the United Nations (UN) Sustainable Development Goals (SDGs), specifically the Universal Health Coverage (UHC) target [2, 3]. Achieving UHC involves all people having access to needed effective health care services with full financial protection by 2030 [4–6]. People are exposed to financial risks or undue financial hardships when the out-of-pocket (OOP) health care expenses incurred are catastrophic or impoverishing [7]. Health care payments are considered catastrophic or impoverishing if household-level OOP expenditure exceeds a predefined threshold of the household’s available resources and a poverty line, respectively [8–10]. Notably, some people may be unable to access health care services because these are not affordable. Hence, non-use of health care may also indicate financial hardships in accessing health care [7, 11–13]. Different methods have evolved over the years to measure the presence of financial hardships or the absence of financial risk protection, with the main difference among the approaches being the measure of a household’s available resources to pay for health care [14, 15].

Globally, households experience financial hardships due to OOP health spending, and the problem is the most severe in the low- and middle-income countries (LMICs) [16–19]. Nearly 11.7% (808 million) of the world’s population incur catastrophic health expenditure (CHE), and 1.4% (97 million) of people are pushed into poverty [17, 20]. According to a recent study, South Asia has the highest incidences of CHE and impoverishment, with OOP expenses being especially regressive in Bangladesh and India [18]. The share of OOP in current health expenditure grew from 61% in 2000 to 74% in 2018 in Bangladesh, the sixth-highest globally and the second-highest in South Asia [21].

Bangladesh’s growing reliance on OOP financing for health care is a major impediment to ensuring financial protection for its people and achieving UHC and the SDGs. Analyzing Bangladesh Household Income and Expenditure Survey (HIES) data, existing studies indicate a lack of financial protection in accessing health care, with CHE and impoverishment incidences ranging from 13.9%-16.2%, and 3.2–3.5%, respectively [22–25]. While Bangladesh HIES collects information on OOP expenses in two separate modules (health and consumption) that differ substantially in magnitude, these are the estimates derived from analyzing data from just one (sometimes without reference to which module’s data were utilized). Additionally, these studies either lacked data recency or used a single round of data. All the studies used the traditional budget-share and capacity-to-pay (CTP) methods [8, 9, 26] despite their documented limitations for policy actions [27, 28]. Also, methodological variations in earlier analyses complicated the generalization of the findings. Like the vast majority of financial risk protection studies worldwide, earlier studies in Bangladesh assessed how the health system protects its citizens by evaluating the consequences of *paying* for health care without accounting for those who required health care but could not afford it. Therefore, the extent to which Bangladeshi households forgo health care due to financial hardships remains unanswered.

In this study, we aim to examine the changes in the level and distribution of financial risk protection in Bangladesh over time. Here, we presented the most updated evidence of recent trends and socioeconomic inequalities in CHE and impoverishment using the latest three rounds of HIES following the normative food, housing (rent), and utilities method [29]. By addressing the limitations of the traditional approaches, this method has been shown to have the best equity and policy implications among different measurement methods of financial hardships [27–29]. The main innovation we introduced to the method is the further decomposition of the non-spending households by reasons to identify the households that forgo care due to financial reasons. Earlier studies emphasized the need to incorporate the cost barrier in assessing financial hardship indicators pointing out that failure to do so will leave the financial hardship indicators narrowly conceived [11, 12, 28, 29]. The new actionable evidence generated from this study will enable Bangladesh and other LMICs that rely heavily on OOP finances to formulate policies that ameliorate financial risk protection in health care and break the nexus between ill-health and poverty.

## Methods

### Data sources

Our study used the latest three rounds of Bangladesh HIES: 2005, 2010, and 2016 with sample sizes of 10,080, 12,240, and 46,076 households, respectively [30]. The HIES is a nationally representative repeated cross-sectional survey undertaken (approximately every five years) by the Bangladesh Bureau of Statistics to assess people’s living standards and poverty levels. HIES 2005 and 2010 samples were drawn using a two-stage stratified random sampling design, while the HIES 2016 sample was drawn using a stratified two-stage cluster sampling technique. Each round of HIES offers two alternative data sources on OOP payments: the consumption module and the health module. The former presents household-level OOP expenses with a 12-month recall period. The latter, on the other hand, provides individual-level OOP expenses with a 30-day recall period for all types of care (inpatient, outpatient) in all rounds except a 12-month recall for inpatient expenses in HIES 2016. The health module collects additional information on illness occurrence, care-seeking behavior, and reasons if ill individuals forgo care (**Box 1**).

**Box 1. Example questions regarding an individual’s health status and care-seeking behavior in the HIES health module**

“Have you suffered from any chronic illness/ disability in the last 12 months?”

“What chronic illness/ disability are you suffering from?’

“Have you suffered from any symptoms of illness/ injury in the last 30 days?”

If yes, “What symptoms/ diseases did you suffer from?”

“Have you sought any medical treatment related to the health problems you suffered in the last 30 days?”

If no, “Why did you not seek any treatment?”

### Measurement of financial risk protection indicators

We defined the CHE and impoverishment indicators following the normative food, housing (rent), and utilities method developed by the WHO Barcelona Office for Health Systems Strengthening [31]. Specifically, we defined household CTP for health care as total consumption expenditure (C) minus an estimated amount for basic needs, called subsistence expenditure (SE). Household SE was estimated using food, rent, and utilities (electricity, gas/fuel, water) expenses among households within 25^th^ to 35^th^ percentiles of the per equivalent person distribution of consumption expenditure. We excluded expenses on tobacco and tobacco-related consumption and dining out to arrive at spending on basic food and used the standard WHO household equivalence scale [26]. Households with total consumption expenditures less than SE (and hence a negative CTP) were categorized as “poor”.

#### Impoverishment effect and non-spenders due to financial reasons

**Fig 1:**
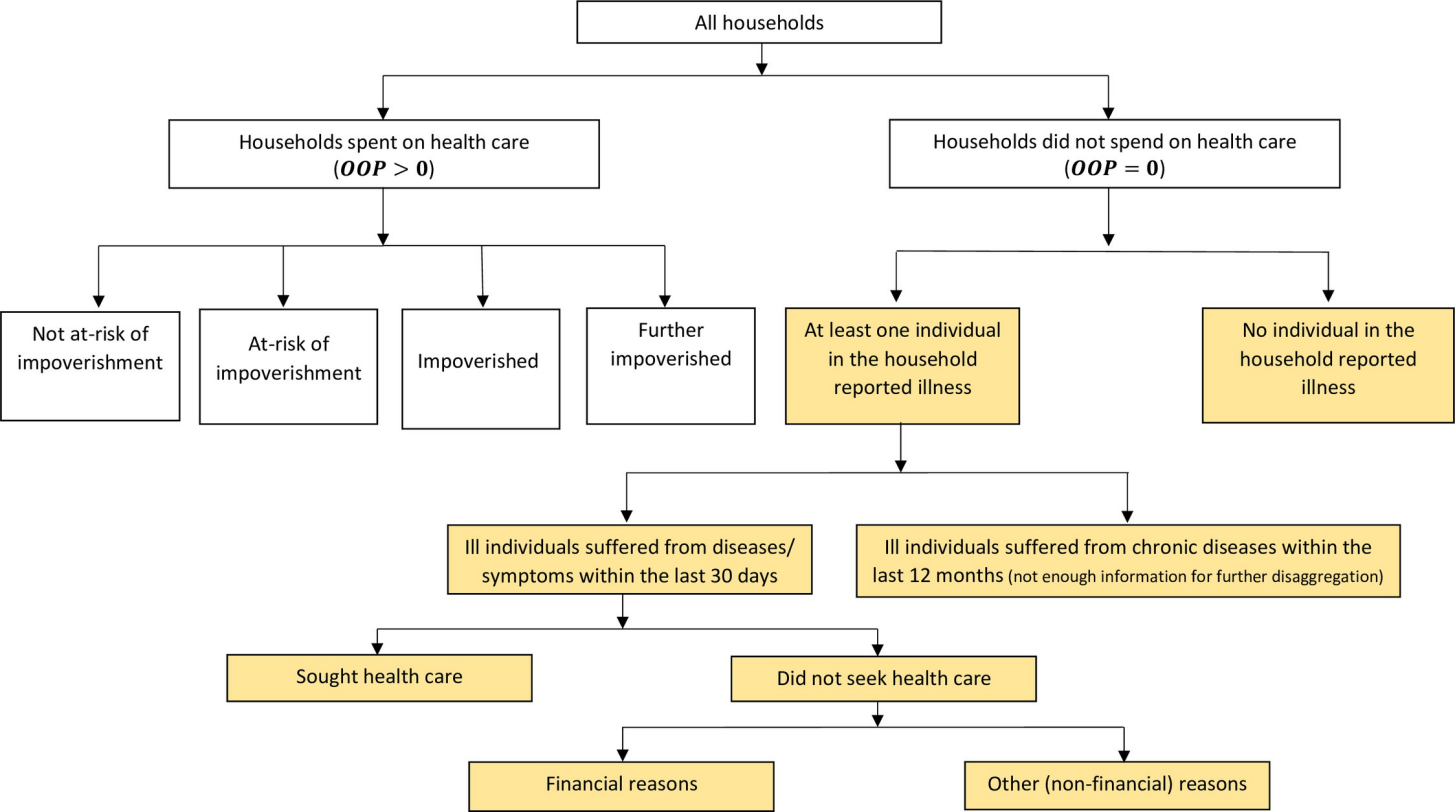
Conceptual framework of impoverishment effect of out-of-pocket (OOP) payments and forgone care due to financial reasons. All households are divided into households with and without OOP payments. Risk categories of impoverishment due to OOP payments are shown on the left-hand side of the diagram. The highlighted right-hand side of the diagram shows the categories of households with zero OOP expenses by reasons.

We used a conceptual framework (Fig 1) for identifying households by their risk of impoverishment due to seeking care and paying from OOP and those who had to refrain from seeking care due to the need to pay from OOP. First, we divided all households into spenders (*OOP*>0) and non-spenders (*OOP* = 0). Then we partitioned the spenders into four non-overlapping categories based on their position around the SE line before and after paying for health care:

*Further impoverished households*. Households were below the SE line (i.e., poor) before OOP payments and slid further below the SE line after OOP payments.

*Impoverished households*. Households above the SE line (i.e., non-poor) before making OOP payments but below the SE line (i.e., poor) after OOP payments.

*Households at-risk of impoverishment*. Households remained above the SE line before and after paying for health care (i.e., non-poor). However, their position was close (within 120%) to the SE line after OOP payments (i.e., near-poor) [32].

*Households not at-risk of impoverishment*. Households stayed above the SE line before and after paying for health care (i.e., non-poor). Their position was way above (more than 120%) the SE line after OOP payments [32].

Non-spenders, on the other hand, have zero OOP expenses and, as such, do not experience catastrophic or impoverishing consequences. However, the households that forgo care due to financial reasons (such as high/ unaffordable treatment costs) also lack financial protection. To identify these households, we disaggregated the non-spenders into the following mutually exclusive categories:

Non-spenders and well

Non-spenders with chronic illness within the last 12 months

Non-spenders due to financial reasons (for diseases/symptoms within the last 30 days)

Non-spenders due to other (non-financial) reasons (for diseases/symptoms within the last 30 days), and

Non-spenders but sought health care (for diseases/symptoms within the last 30 days)

We present further details of each category, including how we converted individual-level information on forgone care to household-level and their alternative definitions, in S1 Table.

#### Catastrophic health expenditure

**Fig 2:**
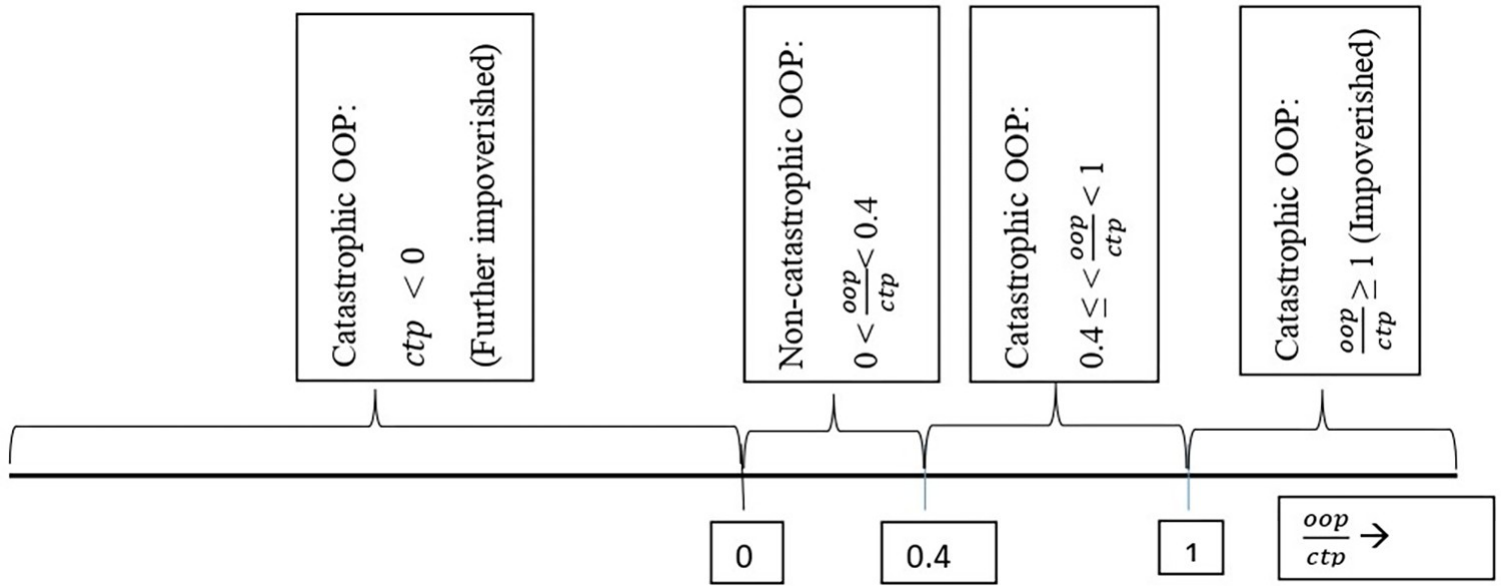
Out-of-pocket health expenditure as a fraction of capacity-to-pay, and catastrophic health expenditure. ctp = capacity-to-pay, oop = out-of-pocket payment.

A household’s OOP expenditure was considered catastrophic if it exceeded 40% of the household’s CTP [32]. Additionally, poor households, because of their inability to meet basic needs (i.e., C < SE *and thus*, *C*−*SE* = *CTP*<0), were also counted as experiencing CHE if they incurred any OOP expenditures (Fig 2). Mathematically,

$$CHE=1\;if\;(\frac{OOP}{CTP}\geq 0.4)\;or\;(OOP>0\;\&\frac{OOP}{CTP}<0)$$

$$CHE=0\;if\;(0\leq \frac{OOP}{CTP}<0.4)$$

Results for 30% and 20% thresholds were also examined.

### Data analysis

After data cleaning, our final samples for 2005, 2010, and 2016 were 10,076, 12,237, and 45,976 households, respectively. Besides measuring the incidences of CHE, impoverishment, and forgone care due to financial reasons, we investigated the distribution of these estimates across consumption quintiles, area of residence, household head’s sex, and education level, and the presence of individuals with chronic illness in households. We used annualized household OOP expenses data from the health module, both as a separate variable and as a component of total consumption expenditure. All expenses in Bangladeshi taka (BDT) were expressed in 2016 prices using the consumer price index (CPI) and then converted into US dollars using the average 2016 exchange rate (USD 1 = BDT 78.468) [33, 34]. We evaluated the robustness of our findings by altering the thresholds used for CHE and impoverishment measurements. Since CHE is the official SDG indicator, we also examined the levels and distributions of CHE using the conventional budget share and CTP methods used in global and regional UHC monitoring (the 10, and 25% of the budget share, 40% of actual non-food expenditure, and 40% normative food expenditure methods) [6, 8, 9, 26]. All results are survey estimates obtained through the survey commands of Stata version 14.2. The Murdoch University Human Research Ethics Committee, Australia granted ethics waiver for this study, given secondary nature of the data used.

We found that annual OOP expenditure differs substantially between the health and consumption modules in each HIES round, potentially resulting in different financial protection statuses for a given household and, thus, different levels and distributions of the incidence of financial protection indicators across all households in the same year. To verify, we repeated all the calculations in two alternative approaches depending on the source of OOP expenses data. In the first approach, OOP expenses originate from the health module; and in the second approach, OOP comes from the consumption module. However, in both scenarios, the OOP spending component of total consumption expenditure is derived from the consumption module. These results are available in the supplementary files.

## Findings

### OOP expenditure

Table 1 presents the level and distribution of mean annual household OOP payments in absolute terms and as a fraction of household consumption expenditure and CTP in 2005, 2010, and 2016. The mean yearly OOP expenditure was USD 115.6 in 2005, which increased by 1.4 times in 2010 (USD 162.1) and 2.1 times in 2016 (USD 242.9). Medicines accounted for nearly two-thirds of total OOP payments in 2005 but almost three-quarters in 2016. During the study period, households in the lowest quintile spent only about 5% to 6% of what the households in the highest quintile spent on health care (USD 17.7 vs. USD 365.2 in 2005; USD 27.8 vs. USD 356.2 in 2010, and USD 42.6 vs. USD 703.2 in 2016). Households with chronic illness had around two times higher OOP than those without the condition in 2005 and 2010 (USD 156.7 vs. USD 83.1 and USD 207.2 vs. USD 88.4, respectively), but more than four times as much in 2016 (USD 411.0 vs. USD 92.1).

**Table 1:** The levels and distributions of out-of-pocket (OOP) expenditure (in absolute and relative terms).

	2005 (n = 10,075)		2010 (n = 12,237)		2016 (n = 45,976)	
**Panel A: Mean annual OOP expenditure (USD) ^a^**						
Total OOP expenditure	115.6	(5.7)	162.1	(11.9)	242.9	(6.5)
**Components**						
consultation	10.9	(0.7)	10.3	(0.5)	15.1	(1.2)
medicine	74.9	(2.8)	93.1	(7.3)	178.4	(4.7)
diagnosis	13.5	(1.9)	17.5	(1.2)	35.0	(1.5)
hospitalization	7.2	(1.2)	10.8	(2.4)	7.6	(0.6)
maternity	3.5	(0.9)	3.5	(1.1)	1.6	(0.3)
contraceptives	n/a	n/a	n/a	n/a	2.3	(0.1)
immunization	n/a	n/a	n/a	n/a	3.0	(1.7)
tips	1.3	(0.8)	2.5	(1.9)	0.6	(0.1)
other charges	4.4	(0.5)	5.7	(0.9)	0.9	(0.1)
**Consumption quintiles**						
lowest	17.7	(0.9)	27.8	(1.5)	42.6	(1.1)
2nd	34.2	(1.5)	49.1	(2.4)	88.4	(2.2)
3rd	64.1	(2.9)	80.5	(4.0)	142.1	(4.2)
4th	97.1	(4.3)	134.1	(6.1)	238.6	(7.7)
highest	365.2	(26.9)	425.8	(50.8)	703.2	(27.7)
**Area of residence**						
rural	107.1	(6.6)	157.6	(14.0)	242.1	(7.5)
urban	140.7	(10.8)	104.9	(9.5)	245.1	(12.9)
**Sex of household head**						
male	119.1	(6.2)	145.5	(11.9)	249.2	(7.0)
female	86.0	(10.3)	130.8	(14.2)	202.5	(9.9)
**Education level of household head**						
no education	91.2	(5.3)	116.2	(6.3)	201.4	(7.1)
below secondary	137.3	(13.9)	173.4	(27.6)	245.7	(8.7)
secondary and above	159.3	(16.2)	175.0	(23.0)	351.0	(19.1)
**Presence of chronic illness**						
no	83.1	(7.5)	88.4	(5.3)	92.1	(5.2)
yes	156.7	(8.6)	207.2	(21.4)	411.0	(10.9)
**Panel B: OOP expenditure as a percentage of total consumption expenditure**						
**Consumption quintile**						
lowest	2.4	(0.1)	2.8	(0.1)	4.7	(0.1)
2nd	3.1	(0.1)	3.2	(0.2)	6.0	(0.2)
3rd	4.2	(0.2)	4.0	(0.2)	7.2	(0.2)
4th	4.6	(0.2)	4.8	(0.2)	8.8	(0.3)
highest	7.8	(0.6)	7.5	(0.9)	12.9	(0.5)
overall	5.7	(0.3)	5.5	(0.4)	9.7	(0.2)
**Area of residence **						
rural	6.1	(0.4)	6.9	(0.6)	10.7	(0.3)
urban	5.0	(0.4)	3.0	(0.3)	7.9	(0.4)
**Sex of household head **						
male	5.8	(0.3)	5.4	(0.4)	9.8	(0.2)
female	5.1	(0.6)	6.3	(0.7)	9.5	(0.4)
**Education level of household head **						
no education	6.0	(0.3)	5.8	(0.3)	10.0	(0.3)
below secondary	6.3	(0.6)	6.5	(1.0)	9.9	(0.3)
secondary and above	4.5	(0.4)	4.0	(0.5)	9.1	(0.5)
**Presence of chronic illness **						
no	4.5	(0.4)	3.7	(0.2)	4.2	(0.2)
yes	6.9	(0.4)	7.2	(0.7)	14.4	(0.3)
**Panel C: OOP expenditure as a percentage of household capacity-to-pay (CTP)**						
**Consumption quintile**						
lowest ^b^	-363.6	(229.3)	-263.6	(127.0)	64.9	(3.7)
2nd	10.6	(0.5)	11.0	(0.5)	15.9	(0.4)
3rd	9.2	(0.4)	8.9	(0.4)	13.7	(0.4)
4th	7.7	(0.3)	8.1	(0.4)	13.6	(0.4)
highest	9.7	(0.7)	9.4	(1.1)	15.7	(0.6)
overall	9.6	(0.4)	9.5	(0.7)	15.4	(0.4)
**Area of residence **						
rural	11.3	(0.6)	13.2	(1.0)	17.8	(0.5)
urban	7.1	(0.5)	4.5	(0.5)	11.5	(0.6)
**Sex of household head **						
male	9.7	(0.5)	9.3	(0.7)	15.5	(0.4)
female	8.3	(1.0)	11.2	(1.2)	14.8	(0.7)
**Education level of household head **						
no education	12.9	(0.7)	12.5	(0.6)	18.3	(0.6)
below secondary	10.2	(0.9)	11.1	(1.6)	15.6	(0.5)
secondary and above	5.9	(0.6)	5.4	(0.7)	12.1	(0.7)
**Presence of chronic illness **						
no	8.0	(0.7)	6.9	(0.4)	7.3	(0.4)
yes	11.0	(0.6)	11.8	(1.1)	21.3	(0.6)

n/a = not available; numbers in parentheses are standard errors.

^a^ All year’s expenses in Bangladeshi taka (BDT) were expressed in 2016 prices using consumer price index, CPI (CPI_2005_ = 69.153, CPI_2010_ = 100, and CPI_2016_ = 152.529) and then converted into US dollars using the 2016 average exchange rate (USD 1 = BDT 78.468).

^b^ CTP = consumption expenditure–subsistence expenditure. Negative values of OOP as a percentage of CTP for the households in the lowest quintile mean that an average household in the bottom quintile had total consumption expenditure less than their respective subsistence expenditure.

OOP expenses increased in relative terms also, from 6% to 10% of household consumption spending and 10% to 15% of household CTP during the study period. In 2005 and 2010, the households in the lowest quintile spent 3.6 and 2.6 times their CTP, respectively, but around two-thirds of CTP in 2016.

### Impoverishment effect

Table 2 shows the impoverishing effects of OOP health payments. Impoverishment incidence showed a clear upward trend during the study period (1.5% to 1.6% to 2.3% in 2005, 2010, and 2016). While further impoverishment incidence increased marginally (with fluctuation) over the study period (3.6% in 2005, 4.1% in 2010, 3.9% in 2016), it was consistently higher than the impoverishment incidence. Over time, households at-risk of impoverishment remained stable at around 5.5%. Lower cut-off values (5% and 10% above subsistence expenditure instead of 20%) produced lower at-risk of impoverishment and higher not at-risk of impoverishment incidences (S2A and S2B Table).

**Table 2:** Incidences of impoverishment effects and non-spenders (including households forgoing care due to financial reasons) (as % of all households).

Risk categories ^a^	2005 (n = 10,075)		2010 (n = 12,237)		2016 (n = 45,976)	
**Spenders**						
1a. Further impoverished	3.6	(0.2)	4.1	(0.2)	3.9	(0.2)
1b. Impoverished	1.5	(0.1)	1.6	(0.1)	2.3	(0.1)
1c. At risk of impoverishment	5.4	(0.2)	5.5	(0.3)	5.5	(0.2)
1d. Not at risk of impoverishment	41.7	(0.5)	41.6	(0.9)	63.0	(0.7)
**2. Non-spenders** ^b^ **(total)**	47.8	(0.5)	47.3	(0.1)	25.3	(0.7)
2a. Non-spender and well	28.4	(0.5)	29.0	(0.9)	20.0	(0.6)
2b. Non-spender with chronic illness in the last 12 months	15.3	(0.4)	17.1	(0.5)	1.6	(0.1)
2c. Non-spender, financial reason, illness in the last 30 days	0.7	(0.1)	0.1	(0.0)	0.3	(0.0)
2c (alt.). Non-spender, financial reason, illness in the last 30 days (alternative definition)	0.8	(0.1)	0.1	(0.0)	0.5	(0.1)
2d. Non-spender, non-financial reasons, illness in the last 30 days	2.7	(0.2)	0.7	(0.1)	2.0	(0.2)
2d (alt.). Non-spender, non-financial reasons, illness in the last 30 days (alternative definition)	2.6	(0.2)	0.7	(0.1)	1.7	(0.2)
2e. Non-spender but sought health care	0.7	(0.1)	0.3	(0.1)	1.4	(0.1)

Households are at risk of impoverishment if consumption after OOP expenditure is between 100% and 120% of subsistence expenditure; numbers in parentheses are standard errors.

^a^ Sum of incidences of risk categories 1a, 1b, 1c, 1d, and 2 = 100%; Sum of incidences of risk categories 1a, 1b, 1c, 1d, 2a, 2b, 2c, 2d, and 2e = 100%; Sum of incidences of risk categories 1a, 1b, 1c, 1d, 2a, 2b, 2c (alt.), 2d (alt.), and 2e = 100%.

### Non-spenders including households forgoing care due to financial reasons

As shown in Table 2, nearly half of all households had zero OOP expenditure in 2005 and 2010, falling to almost a quarter in 2016. Families with no reported illness decreased by a third from around 29.0% in 2005 and 2010 to about 20.1% in 2016, indicating an increase in morbidity between the last two periods. Between 2010 and 2016, the proportion of chronic illness-affected households seeking care increased dramatically, shown by a marked decline in non-spenders with chronic disease(s) from 17.1% to 1.6%. Households that did not seek care because of financial constraints fluctuated over time but remained low at less than 1% (0.7%, 0.1%, 0.3% in 2005, 2010, and 2016, respectively).

#### Distribution of impoverishment effect

In general, households in the lowest quintile, rural areas, with female heads, heads with no education, and households inflicted with chronic illness had higher further impoverishment, impoverishment, and at-risk of impoverishment incidences than their counterparts (S3A–S3E Table). Between 2005 and 2016, impoverishment incidence among the lowest quintile households increased from 5.5% to 8.6%, while it remained unchanged at 0.3% among the highest quintile (S3 Table). Simultaneously, lowest quintile households falling deeper into poverty increased from 18.0% to 19.4%, while those unable to afford care due to high costs decreased from 1.6% to 0.7%.

### CHE and its distribution

Table 3 shows the incidence of CHE at 40% of the household’s CTP threshold. The mean CHE incidence increased from 11.5% in 2005 to 11.9% in 2010, then jumped to 16.6% in 2016. The lowest quintile households experienced the highest CHE incidence throughout the study period among the different quintiles, increasing from 29.5% to 33.2% to 37.6% in 2005, 2010, and 2016, respectively, compared to 7.6% to 7.2% and 13.0% among the households in the highest quintile in the corresponding years. Each year, CHE was higher among households in rural areas, with female heads, with heads having no education, and with chronic illness compared to their respective counterparts. Notably, in 2016, households with chronic illness incurred close to three times the incidence of the households without such conditions (25.0% vs. 9.1%). Lowering the threshold to 30% and 20% of CTP consistently increased the overall CHE incidence. However, its distribution across equity strata remained almost unchanged (S4A and S4B Table).

**Table 3:** The levels and distributions of the incidence of catastrophic health expenditure (%) by year; normative food, housing, and utilities method (40% threshold).

	2005 (n = 10,075)		2010 (n = 12,237)		2016 (n = 45,976)	
**Consumption quintiles**						
lowest	29.5	(1.1)	33.2	(1.3)	37.6	(0.9)
2nd	8.9	(0.7)	8.1	(0.7)	12.3	(0.5)
3rd	6.5	(0.6)	5.6	(0.5)	9.8	(0.5)
4th	4.9	(0.5)	5.2	(0.5)	10.2	(0.6)
highest	7.6	(0.7)	7.2	(1.3)	13.0	(0.7)
overall	11.5	(0.3)	11.9	(0.4)	16.6	(0.4)
**Area of residence**						
rural	12.8	(0.4)	14.4	(0.6)	19.3	(0.5)
urban	7.4	(0.5)	5.1	(0.4)	9.9	(0.6)
**Sex of household head**						
male	11.0	(0.3)	11.1	(0.5)	16.1	(0.4)
female	15.9	(1.2)	16.7	(1.1)	20.0	(0.8)
**Level of education of household head**						
no education	17.4	(0.9)	15.4	(0.7)	20.8	(0.6)
below secondary	8.7	(0.6)	10.1	(0.6)	15.4	(0.6)
secondary and above	4.3	(0.6)	3.9	(0.5)	8.5	(0.6)
**Presence of chronic illness**						
no	9.7	(0.4)	10.3	(0.5)	9.1	(0.4)
yes	13.7	(0.5)	13.7	(0.6)	25.0	(0.6)

Numbers in parentheses are standard errors

CHE is defined as household OOP expenditure exceeding 40% of household capacity-to-pay plus any health expenditure by poor households. Therefore, the overall incidence of CHE does not reflect the average of the CHE incidences of the five consumption quintiles

### CHE and its distribution using traditional methods

The use of conventional budget share and CTP methods also revealed deteriorations in financial protection (Tables 4 and 5). The 10% of budget share method (the official SDG indicator to track progress towards achieving UHC) showed that CHE incidence doubled during the study period, climbing from 12.5% in 2005 to 24.7% in 2016. This upward trend persisted across all equity strata. However, CHE was concentrated among the households in the highest quintile when measured using the budget-share (Table 4) and the actual food expenditure method (Table 5). However, the normative food expenditure method, showed a higher concentration of CHE among households with low economic status (Table 5).

**Table 4:** The levels and distributions of the incidence of catastrophic health expenditure (%) by year; budget share method (SDG indicator 3.8.2).

	Threshold: OOP > 10% of consumption expenditure						Threshold: OOP > 25% of consumption expenditure					
	2005 (n = 10,075)		2010 (n = 12,237)		2016 (n = 45,976)		2005 (n = 10,075)		2010 (n = 12,237)		2016 (n = 45,976)	
**Consumption quintiles **												
lowest	6.6	(0.6)	8.0	(0.7)	16.2	(0.5)	0.8	(0.2)	1.2	(0.2)	3.3	(0.2)
2nd	9.7	(0.7)	9.5	(0.7)	20.5	(0.7)	1.6	(0.3)	1.6	(0.3)	5.1	(0.3)
3rd	13.1	(0.8)	12.8	(0.8)	24.3	(0.8)	3.6	(0.4)	2.8	(0.4)	7.4	(0.4)
4th	14.6	(0.9)	15.8	(0.9)	29.0	(1.0)	4.5	(0.5)	4.7	(0.5)	11.0	(0.6)
highest	18.6	(1.0)	17.8	(1.2)	33.3	(1.3)	9.1	(0.7)	8.8	(0.7)	16.8	(0.8)
overall	12.5	(0.4)	12.8	(0.5)	24.7	(0.5)	3.9	(0.2)	3.8	(0.2)	8.7	(0.2)
**Area of residence**												
rural	12.8	(0.4)	14.5	(0.6)	26.3	(0.5)	3.9	(0.2)	4.4	(0.3)	9.4	(0.3)
urban	11.7	(0.7)	8.1	(0.7)	20.4	(1.2)	4.1	(0.4)	2.4	(0.3)	6.9	(0.4)
**Sex of household head**												
male	12.5	(0.4)	12.4	(0.5)	24.5	(0.6)	3.9	(0.2)	3.6	(0.2)	8.6	(0.3)
female	12.9	(1.1)	15.2	(1.0)	25.6	(0.9)	4.1	(0.7)	5.2	(0.6)	9.6	(0.6)
**Level of education of household head**												
no education	14.0	(0.9)	13.0	(0.6)	24.9	(0.6)	4.2	(0.5)	4.0	(0.3)	8.8	(0.3)
below secondary	13.4	(0.7)	14.1	(0.7)	25.0	(0.6)	4.3	(0.4)	3.9	(0.4)	8.8	(0.4)
secondary and above	10.2	(0.9)	9.5	(0.8)	23.0	(1.1)	4.1	(0.6)	3.2	(0.4)	8.3	(0.7)
**Presence of chronic illness**												
no	9.3	(0.4)	8.9	(0.5)	10.5	(0.5)	2.9	(0.2)	2.7	(0.2)	3.1	(0.2)
yes	16.6	(0.6)	17.3	(0.7)	40.4	(0.7)	5.3	(0.4)	5.1	(0.4)	15.0	(0.4)

Numbers in parentheses are standard errors

**Table 5:** The levels and distributions of the incidence of catastrophic health expenditure (%) by year; capacity-to-pay methods (actual food expenditure, and normative food expenditure methods).

	Threshold: OOP > 40% of total non-food expenditure						Threshold: OOP ≥ 40% of subsistence expenditure (based on standard food expenditure)					
	2005 (n = 10,075)		2010 (n = 12,237)		2016 (n = 45,976)		2005 (n = 10,075)		2010 (n = 12,237)		2016 (n = 45,976)	
**Consumption quintiles **												
lowest	4.1	(0.5)	4.4	(0.5)	8.0	(0.4)	11.4	(0.8)	12.6	(0.8)	18.3	(0.6)
2nd	5.7	(0.5)	4.9	(0.5)	8.9	(0.4)	8.9	(0.7)	8.1	(0.7)	12.3	(0.5)
3rd	7.7	(0.6)	6.7	(0.6)	10.7	(0.5)	6.5	(0.6)	5.6	(0.5)	9.8	(0.5)
4th	7.7	(0.6)	7.8	(0.6)	13.6	(0.8)	4.9	(0.5)	5.2	(0.5)	10.2	(0.6)
highest	10.2	(0.7)	10.7	(0.8)	16.8	(0.9)	7.6	(0.7)	7.2	(0.7)	13.0	(0.7)
overall	7.1	(0.3)	6.8	(0.3)	11.6	(0.3)	7.8	(0.3)	7.8	(0.3)	12.7	(0.3)
**Area of residence**												
rural	7.5	(0.3)	8.0	(0.4)	13.0	(0.3)	8.5	(0.4)	9.2	(0.4)	14.5	(0.4)
urban	5.8	(0.5)	3.5	(0.3)	8.0	(0.5)	5.9	(0.5)	3.8	(0.3)	8.2	(0.5)
**Sex of household head**												
male	7.0	(0.3)	6.5	(0.3)	11.5	(0.3)	7.8	(0.3)	7.6	(0.3)	12.7	(0.3)
female	7.5	(0.9)	8.3	(0.8)	12.5	(0.7)	8.4	(1.0)	9.0	(0.8)	12.9	(0.6)
**Level of education of household head**												
no education	9.4	(0.7)	7.5	(0.4)	13.0	(0.4)	10.9	(0.8)	9.2	(0.5)	14.8	(0.5)
below secondary	6.8	(0.5)	6.8	(0.5)	11.2	(0.4)	6.9	(0.5)	7.7	(0.5)	12.4	(0.4)
secondary and above	4.9	(0.6)	4.1	(0.5)	8.9	(0.7)	4.1	(0.6)	3.3	(0.4)	7.8	(0.6)
**Presence of chronic illness**												
no	5.4	(0.3)	4.9	(0.3)	4.6	(0.3)	6.3	(0.4)	6.2	(0.4)	5.8	(0.3)
yes	9.2	(0.5)	8.9	(0.5)	19.4	(0.5)	9.8	(0.5)	9.5	(0.5)	20.4	(0.5)

Numbers in parentheses are standard errors

We verified all results reported here (Tables 1–5) using alternative calculations outlined in the methods section) and found broadly consistent patterns, despite magnitude differences (S5–S7, S8A–S8D Tables).

## Discussion

Analyzing the latest three rounds of Bangladesh HIES data (2005, 2010, and 2016), we found that OOP expenditure and its consequent catastrophic and impoverishing effects increased over the years. The proportion of households forgoing health care due to financial constraints remained relatively small. The households in the lowest quintile were the least financially protected throughout the study period. In recent years, chronic disease has also emerged as a significant barrier to financial protection.

The rise in OOP expenditure is linked to the diminishing government share in Bangladesh’s current health expenditure (from 22% to 18% between 2005 and 2016) [21]. Additionally, the country’s continuous economic growth has increased the population’s purchasing power [35]. Rising income levels and education have led people to seek more and better-quality care, resulting in higher OOP health expenditures [36]. Between 2010 and 2016, we found a sharp increase in morbidity and a drastic fall in non-spending households, which explains the substantial increase in OOP expenses during that period. A large body of literature suggests that high OOP expenditures are positively associated with CHE, impoverishment, and the adoption of coping strategies [37–41]. Accordingly, our study found an increasing trend of CHE, irrespective of the measurement methods applied, where the absolute level of CHE was substantial in 2016. The rising trend of CHE indicates Bangladesh is not well-positioned in achieving UHC and, therefore, the SDGs by 2030.

Between 2005 and 2016, poverty attributable to OOP expenditures also increased from 1.5% to 2.3% or 2.1 to 3.6 million people at the population level. Further impoverishment was a more severe problem, growing from 3.6% to 3.9%, or 5.0 to 6.2 million individuals. Additionally, more than 7 million people were at risk of impoverishment throughout the study period. However, during the same period, the national headcount poverty rate declined remarkably from 40.0% to 24.3%, implying that overall poverty alleviation was not accompanied by improved financial protection in health care in Bangladesh [42]. The increasing trend in impoverishment in Bangladesh contrasts with the declining trends in some other LMICs in Asia and Africa [43–47]. The common feature of these countries is an implementation of health financing reform by introducing or expanding publicly funded health protection schemes.

The incidence of non-spenders due to financial reasons, however, remained relatively low (≤1%) throughout the study period. This result is not surprising because people tend to consume health care even if it means dis-saving, borrowing, and selling assets [48–50]. The increased dependency on low-cost, informal health care providers over the years (from about 50% in 2010 to 70% in 2016) could be another explanation [42, 51].

Although financial protection was disparate across all the equity strata examined, the lowest quintile households were the least protected throughout the study period. These results align with previous studies in Bangladesh and other LMICs [22, 41, 43–45, 52–55]. The absence of adequate safety-net programs and risk pooling mechanisms for health care is to blame for the afflicting financial risk protection outcomes for the people in Bangladesh [56].

The disparity in CHE incidence was also remarkable between households with and without chronic illness, which widened over time. Like other LMICs, Bangladesh is undergoing rapid demographic and epidemiological transitions, resulting in population aging and an increasing burden of chronic non-communicable diseases [57–59]. Because chronic illnesses necessitate lengthy and usually expensive treatment, affected households seeking care are more likely to experience financial hardships [60–62]. The dramatic increase in the proportion of chronic illness-affected households seeking care between 2010 and 2016 explains the jump in CHE among these households in 2016. Previous studies in rural settings also identified chronic illnesses as significant drivers of financial hardships in Bangladesh [63–66].

Given the continued actual and projected dependence on OOP expenditure and diminishing government share in total health expenditure, it appears that achieving UHC and, thus, the SDGs by 2030 will be challenging for Bangladesh [67, 68]. The Health Care Financing Strategy (HCFC) 2012–2032, the country’s statutory roadmap to UHC, outlines a plan to reduce OOP expenses primarily by introducing social health protection schemes for the poor and the formal sector employees, with a gradual move to the social health protection scheme for the entire population [56]. In 2016, the government introduced a tax-funded, 100% subsidized social health protection scheme, *Shasthyo Suroksha Karmasuchi (SSK)*, for the below-poverty line population in three sub-districts of Dhaka [69]. The scheme covers inpatient care for 50+ disease groups, with a benefit of USD 620 per household per year [69]. SSK is still in the piloting phase, and its financial protection effects are yet to be known. Social health insurance for the formal sector has not been rolled out yet.

The Bangladesh government recognizes chronic noncommunicable diseases (NCDs) as a public health challenge and has made significant strides in the policy environment to prevent and control such diseases [70]. Notables are the policies on tobacco taxes, cigarette packaging, alcohol advertising, and the Multisectoral Action Plan for Prevention and Control of NCDs, 2018–2025 [71]. However, like the HCFC, these plans and policies also suffer implementation challenges [72]. Additionally, the country’s chronic NCD control budget (US cents 8.2/capita/year) falls far short of what is required to implement the "WHO best buys" in lower-middle-income countries (USD 1.5/capita/year) [70, 73].

Global evidence shows that a higher share of the health budget directed towards government or social health insurance schemes lowers CHE and impoverishment [17, 20, 74]. Therefore, successfully implementing government-funded health protection schemes, carefully designed to address the disproportionate financial burden on the poor and chronically ill, is crucial to achieving financial protection and UHC in Bangladesh. The government needs further focus and transformative measures to successfully implement the HCFS and chronic disease prevention and control policies. The government must increase the health sector budget allocation substantially, maintain it, and manage finances efficiently. Bangladesh could follow other LMICs and use the fiscal liver to secure and sustain additional funds for the health sector (for example, the Philippines ’ "sin taxes" on tobacco and alcohol) [75, 76].

Unlike previous LMIC studies, including Bangladesh, our analysis makes visible the further impoverished and at risk of impoverishment households due to OOP payments and those who required health care but could not afford it. We verified all results by using the two distinct sources of OOP expenditure in HIES. However, it is worth noting that inpatient expenses had a shorter recall period in 2005 and 2010 (30 days) than in 2016 (12 months), which, according to Lu et al. (2009), may result in overestimated annual OOP expenses in 2005 and 2010 [77]. Even so, the deteriorating trend of financial protection remains unchanged.

Overall, financial risk protection in health care deteriorated in Bangladesh between 2005 and 2016. The distribution of financial risk protection was inequitable across all the equity strata examined in this study, with the households in the lowest quintile suffering the worst outcomes. Chronic diseases emerged as a major obstacle to achieving UHC in the latest year. Reversing the deteriorating trends of financial risk protection and addressing the inequities in its distribution will require implementing UHC initiatives and thus providing financial protection against illness.

## Supporting information

S1 Table Household categories according to the risk of impoverishment due to out-of-pocket (OOP) spending on health care including non-spender categories. (DOCX)

S2 Table **a.** Incidences of impoverishment effects and non-spenders (including households forgoing care due to financial reasons) (as % of all households); at risk of impoverishment threshold: between 100% and 110% of subsistence expenditure. **b.** Incidences of impoverishment effects and non-spenders (including households forgoing care due to financial reasons) (as % of all households); at risk of impoverishment threshold: between 100% and 105% of subsistence expenditure.
(DOCX)

S3 Table **a.** Incidences of impoverishment effects and non-spenders (including households forgoing care due to financial reasons) (as % of all households) by consumption quintile. **b.** Incidences of impoverishment effects and non-spenders (including households forgoing care due to financial reasons) (as % of all households) by area of residence. **c.** Incidences of impoverishment effects and non-spenders (including households forgoing care due to financial reasons) (as % of all households) by sex of household head. **d.** Incidences of impoverishment effects and non-spenders (including households forgoing care due to financial reasons) (as % of all households) by household head’s education level. **e.** Incidences of impoverishment effects and non-spenders (including households forgoing care due to financial reasons) (as % of all households) by the presence of chronic illness.
(DOCX)

S4 Table **a.** Incidence of catastrophic health expenditure (%) over time by equity strata; normative food, rent, and utilities method (threshold: OOP≥ 30% of CTP). **b.** Incidence of catastrophic health expenditure (%) over time by equity strata; normative food, rent, and utilities method (threshold: OOP≥ 20% of CTP).
(DOCX)

S5 Table The levels and distributions of out-of-pocket (OOP) expenditure (alternative calculations). (DOCX)

S6 Table Incidences of impoverishment effects and non-spenders (alternative calculations). (DOCX)

S7 Table The levels and distributions of the incidence of catastrophic health expenditure (%); normative food, housing, and utilities method, 40% threshold (alternative calculations). (DOCX)

S8 Table **a.** The levels and distributions of the incidence of catastrophic health expenditure (%) by year; budget share method, 10% threshold (alternative calculations). **b.** The levels and distributions of the incidence of catastrophic health expenditure (%) by year; budget share method, 25% threshold (alternative calculations). **c.** The levels and distributions of the incidence of catastrophic health expenditure (%) by year; actual food expenditure method, 40% threshold (alternative calculations). **d.** The levels and distributions of the incidence of catastrophic health expenditure (%) by year; normative food expenditure method, 40% threshold (alternative calculations).
(DOCX)
