# Supplementary material for: Financial risk protection in health care in Bangladesh in the era of Universal Health Coverage
Source: PLoS One. 2022 Jun 24;17(6):e0269113. doi: 10.1371/journal.pone.0269113 (PMC9231789; doi:10.1371/journal.pone.0269113)
Supplement: S1 Table — (DOCX) [file pone.0269113.s001.docx]

**Article title:** Financial risk protection in Bangladesh in the era of Universal Health Coverage

**Journal name:** *PLOS ONE*

**S1 Table: Household categories according to the risk of impoverishment due to out-of-pocket (OOP) spending on health care including non-spender categories**

| **Risk categories** | **Definition** |
| --- | --- |
| 1. **Spenders** | Household-level OOP is positive ($oop>0).$ |
| *1a. Further impoverished* | Households that did not have enough resources to meet basic needs and thus, had a negative CTP (c<se; ctp<0) were deemed poor. Any health expenditure made them further impoverished (oop>0; $\frac{oop}{ctp}<0)$. |
| *1b. Impoverished* | OOP payments impoverished a non-poor household if the household was above the SE line before paying for health care but found itself below the SE line after paying for health care [c≥se; ctp≥0; (c-oop)≤se; $\frac{oop}{ctp}\geq1$ ]. |
| *1c. At-risk of impoverishment* | OOP payments left (non-poor) households above but close to the (within 120% of the) basic needs line  [ c>se; ctp>0; se < (c-oop) ≤ 1.2*se; $0<\frac{oop}{ctp}<1$]. |
| *1d. Not at-risk of impoverishment* | OOP payments did not bring (non-poor) households close to the basic needs line  [c>se; ctp>0; (c-oop) > 1.2*se; $0<\frac{oop}{ctp}<1$ ]. |
| 1. **Non-spenders** | Households that had zero OOP payments (oop=0; $\frac{oop}{ctp}=$0) were non-spenders. (As an extension of the original normative food, housing (rent), and utilities method, we divided non-spending households into another five mutually exclusive groups by utilizing information on illness and care-seeking as follows) |
| *2a. Non-spenders and well* | Households did not have any individual suffering from any disease/ symptom within the last 30 days (n_ill_30d) or any chronic illness within the last 12 months preceding the survey (n_ill_12m):  $oop=0 \& n\_ill\_30d=0 \& n\_ill\_12m=0$  It is important to note that, the disease categories for the last 30 days and the last 12 months are different: The diseases/ symptoms list for the last 30 days recall period consists of both chronic and non-chronic health problems and hence includes a broader range of diseases (but not all, e. g. diabetes) compared to the chronic diseases specified for the last 12-month duration. Therefore, having no ill individual with chronic illness in the last 12 months does not necessarily mean that the household had no ill individuals in the last 30 days and vice-versa. |
| *2b.* *Non-spenders with chronic illnesses within the last 12 months* | These households had individuals with chronic illness within the last 12 months preceding the survey but reported no health expenditure: $oop=0 \& \& n\_ill\_12m \geq1$  No information was available to ascertain if these households faced financial or other barriers or if they sought care but did not have to pay. |
| *2c.* *Non-spenders due to financial reasons (for illnesses within the last 30 days)* | These are the households with individuals suffering from any disease/ symptom within the last 30 days preceding the survey that reported zero OOP expenses because health care was not sought. Additionally, all the individuals in the household who forwent care did so because of financial reasons implying the number of individuals forgoing care $(n\_forgo\_care)$ within a household equaled the number of individuals mentioning financial barrier $(n\_forgo\_fin)$ as the reason:  $oop=0 \& n\_ill\_30d\geq1 \& n\_forgo\_care\geq1 \& n\_forgo\_care=n\_forgo\_fin$  $\boldsymbol{2}\boldsymbol{c}\left( \boldsymbol{alt.} \right)\boldsymbol{. Alternative definition:}$  In an alternative, less stringent definition, a household was in this category if at least one individual did not seek care due to financial reasons. This implies that both $n\_forgo\_care$ and $n\_forgo\_fin$ were greater or equal to one but were not necessarily equal.  $oop=0 \& n\_ill\_30d\geq1 \& n\_forgo\_care\geq1 \& n\_forgo\_fin\geq1$ |
| *2d.* *Non-spenders due to other (non-financial) reasons (for illnesses within the last 30 days)* | Non-spending households in this category had at least one individual in the household who did not seek care for their health problems that occurred within the last 30 days for other non-financial reasons.  $oop=0 \& n\_ill\_30d\geq1 \& n\_forgo\_care\geq1 \& n\_forgo\_fin\geq0 \& n\_forgo\_care\neq n\_forgo\_fin$  $\boldsymbol{2}\boldsymbol{d}\left( \boldsymbol{alt.} \right)\boldsymbol{. Alternative definition:}$  In an alternative definition, households were in this category if all individuals forgoing health care mentioned other (non-financial) barriers as the reason.  $oop=0 \& n\_ill\_30d\geq1 \& n\_forgo\_care\geq1 \& n\_forgo\_fin=0$ |
| *2e.* *Non-spenders but sought health care (for illnesses within the last 30 days)* | Individuals in these households suffered from disease(s)/ symptom(s) within the last 30 days, sought health care but had zero OOP.  $oop=0 \& n\_ill\_30d\geq1 \& n\_forgo\_care=0$ |

OOP = Out-of-pocket

- Sum of incidences of risk categories 1 and 2 =100%
- Sum of incidences of risk categories 1a, 1b, 1c, 1d, and 2 = 100%
- Sum of incidences of risk categories 1a, 1b, 1c, 1d, 2a, 2b, 2c, 2d, and 2e = 100%
- Sum of incidences of risk categories 1a, 1b, 1c, 1d, 2a, 2b, 2c (alt.), 2d (alt.), and 2e = 100%
