# Supplementary material for: Financial risk protection in health care in Bangladesh in the era of Universal Health Coverage
Source: PLoS One. 2022 Jun 24;17(6):e0269113. doi: 10.1371/journal.pone.0269113 (PMC9231789; doi:10.1371/journal.pone.0269113)
Supplement: S3 Table — a. Incidences of impoverishment effects and non-spenders (including households forgoing care due to financial reasons) (as % of all households) by consumption quintile. b. Incidences of impoverishment effects and non-spenders (including households forgoing care due to financial reasons) (as % of all households) by area of residence. c. Incidences of impoverishment effects and non-spenders (including households forgoing care due to financial reasons) (as % of all households) by sex of household head. d. Incidences of impoverishment effects and non-spenders (including households forgoing care due to financial reasons) (as % of all households) by household head’s education level. e. Incidences of impoverishment effects and non-spenders (including households forgoing care due to financial reasons) (as % of all households) by the presence of chronic illness. (DOCX) [file pone.0269113.s003.docx]

**Article title:** Financial risk protection in Bangladesh in the era of Universal Health Coverage

**Journal name:** *PLOS ONE*

**S3a Table: Incidences of impoverishment effects and non-spenders (including households forgoing care due to financial reasons) (as % of all households) by consumption quintile**

| **Risk categories^a^** |  | | | | | |
| --- | --- | --- | --- | --- | --- | --- |
|  | **2005**  **(n=10,075)** | | **2010**  **(n=12,237)** | | **2016**  **(n=45,976)** | |
|  | **poorest** | **richest** | **poorest** | **richest** | **poorest** | **Richest** |
| 1. **Spenders** |  |  |  |  |  |  |
| 1a. Further impoverished | 18.0  (0.9) | n/a  (n/a) | 20.6  (1.1) | n/a  (n/a) | 19.4  (0.6) | n/a  (n/a) |
| 1b. Impoverished | 5.5  (0.6) | 0.3  (0.1) | 6.0  (0.5) | 0.1  (0.1) | 8.6  (0.4) | 0.3  (0.1) |
| 1c. At risk of impoverishment | 17.5  (0.9) | 0.4  (0.2) | 18.4  (1.1) | 0.3  (0.1) | 21.1  (0.6) | 0.3  (0.1) |
| 1d. Not at risk of impoverishment | 0.6  (0.2) | 59.5  (1.2) | 1.0  (0.2) | 55.7  (1.9) | 14.7  (0.5) | 84.4  (1.1) |
| **2. Non-spenders (total)** | 58.3  (1.2) | 39.8  (1.2) | 54.0  (1.6) | 43.9  (1.9) | 36.4  (1.0) | 15.1  (1.1) |
| 2a. Non-spender and well | 34.2  (1.1) | 22.7  (1.1) | 34.2  (1.6) | 24.6  (1.7) | 27.8  (0.9) | 11.8  (1.0) |
| 2b. Non-spender with chronic illness in the last 12 months | 18.0  (0.9) | 14.7  (0.9) | 17.9  (1.0) | 18.8  (1.2) | 2.7  (0.2) | 0.9  (0.1) |
| 2c. Non-spender, financial reason, illness in the last 30 days | 1.6  (0.3) | 0.1  (0.1) | 0.4  (0.1) | 0.0  (0.0) | 0.7  (0.1) | 0.0  (0.0) |
| 2c (alt.). Non-spender, financial reason, illness in the last 30 days (alternative definition) | 1.7  (0.3) | 0.1  (0.1) | 0.4  (0.1) | 0.0  (0.0) | 0.7  (0.1) | 0.7  (0.2) |
| 2d. Non-spender, non-financial reasons, illness in the last 30 days | 4.0  (0.5) | 1.9  (0.4) | 1.0  (0.2) | 0.4  (0.2) | 2.7  (0.3) | 1.6  (0.4) |
| 2d (alt.). Non-spender, non-financial reasons, illness in the last 30 days (alternative definition) | 3.8  (0.4) | 1.9  (0.4) | 1.0  (0.2) | 0.4  (0.2) | 2.7  (0.3) | 1.0  (0.2) |
| 2e. Non-spender but sought health care | 0.5  (0.2) | 0.5  (0.2) | 0.5  (0.2) | 0.1  (0.1) | 2.3  (0.2) | 0.6  (0.1) |

Numbers in parentheses are standard errors

^a^ Sum of incidences of risk 1a, 1b, 1c, 1d, and 2 = 100%; Sum of incidences of risk categories 1a, 1b, 1c, 1d, 2a, 2b, 2c, 2d, and 2e = 100%; Sum of incidences of risk categories 1a, 1b, 1c, 1d, 2a, 2b, 2c (alt.), 2d (alt.), and 2e = 100%

**Article title:** Financial risk protection in Bangladesh in the era of Universal Health Coverage

**Journal name:** *PLOS ONE*

**S3b Table: Incidences of impoverishment effects and non-spenders (including households forgoing care due to financial reasons) (as % of all households) by area of residence**

| **Risk categories^a^** |  | | | | | |
| --- | --- | --- | --- | --- | --- | --- |
|  | **2005**  **(n=10,075)** | | **2010**  **(n=12,237)** | | **2016**  **(n=45,976)** | |
|  | rural | urban | rural | urban | rural | urban |
| 1. **Spenders** |  |  |  |  |  |  |
| 1a. Further impoverished | 4.3  (0.3) | 1.5  (0.2) | 5.2  (0.3) | 1.3  (0.2) | 4.7  (0.2) | 1.7  (0.2) |
| 1b. Impoverished | 1.8  (0.2) | 0.6  (0.1) | 1.9  (0.2) | 0.6  (0.1) | 2.8  (0.1) | 1.1  (0.1) |
| 1c. At risk of impoverishment | 6.3  (0.3) | 2.7  (0.3) | 6.5  (0.4) | 2.5  (0.3) | 6.6  (0.2) | 2.7  (0.3) |
| 1d. Not at risk of impoverishment | 41.1  (0.6) | 43.6  (1.1) | 43.8  (1.1) | 35.4  (1.5) | 60.6  (0.8) | 69.6  (1.5) |
| **2. Non-spenders (total)** | 46.4  (0.6) | 51.7  (1.1) | 42.5  (1.2) | 60.2  (1.7) | 25.4  (0.9) | 25.0  (1.4) |
| 2a. Non-spender and well | 27.3  (0.6) | 31.6  (1.0) | 25.6  (1.0) | 38.3  (1.7) | 19.9  (0.7) | 20.5  (1.2) |
| 2b. Non-spender with chronic illness in the last 12 months | 14.8  (0.4) | 16.8  (0.8) | 15.7  (0.6) | 21.2  (1.1) | 1.7  (0.2) | 1.3  (0.2) |
| 2c. Non-spender, financial reason, illness in the last 30 days | 0.8  (0.1) | 0.3  (0.1) | 0.1  (0.0) | 0.1  (0.0) | 0.3  (0.0) | 0.3  (0.1) |
| 2c (alt.). Non-spender, financial reason, illness in the last 30 days (alternative definition) | 0.9  (0.1) | 0.3  (0.1) | 0.8  (0.1) | 0.1  (0.0) | 0.5  (0.1) | 0.5  (0.1) |
| 2d. Non-spender, non-financial reasons, illness in the last 30 days | 2.9  (0.2) | 2.3  (0.3) | 0.8  (0.1) | 0.4  (0.1) | 2.0  (0.2) | 1.9  (0.4) |
| 2d (alt.). Non-spender, non-financial reasons, illness in the last 30 days (alternative definition) | 2.8  (0.2) | 2.2  (0.3) | 0.4  (0.1) | 0.4  (0.1) | 1.7  (0.2) | 1.7  (0.4) |
| 2e. Non-spender but sought health care | 0.7  (0.1) | 0.6  (0.2) | 0.4  (0.1) | 0.2  (0.1) | 1.5  (0.1) | 1.0  (0.2) |

Numbers in parentheses are standard errors

^a^ Sum of incidences of risk categories 1a, 1b, 1c, 1d, and 2 = 100%; Sum of incidences of risk categories 1a, 1b, 1c, 1d, 2a, 2b, 2c, 2d, and 2e = 100%; Sum of incidences of risk categories 1a, 1b, 1c, 1d, 2a, 2b, 2c (alt.), 2d (alt.), and 2e = 100%

**Article title:** Financial risk protection in Bangladesh in the era of Universal Health Coverage

**Journal name:** *PLOS ONE*

**S3c Table: Incidences of impoverishment effects and non-spenders (including households forgoing care due to financial reasons) (as % of all households) by sex of household head**

| **Risk categories^a^** |  | | | | | |
| --- | --- | --- | --- | --- | --- | --- |
|  | **2005**  **(n=10,075)** | | **2010**  **(n=12,237)** | | **2016**  **(n=45,976)** | |
|  | **male** | **female** | **male** | **female** | **male** | **female** |
| 1. **Spenders** |  |  |  |  |  |  |
| 1a. Further impoverished | 3.2  (0.2) | 7.5  (0.8) | 3.5  (0.2) | 7.7  (0.7) | 3.4  (0.2) | 7.1  (0.5) |
| 1b. Impoverished | 1.5  (0.1) | 1.7  (0.5) | 1.5  (0.1) | 2.2  (0.4) | 2.1  (0.1) | 3.8  (0.4) |
| 1c. At risk of impoverishment | 5.5  (0.3) | 4.6  (0.7) | 5.6  (0.3) | 4.7  (0.5) | 5.6  (0.2) | 4.9  (0.3) |
| 1d. Not at risk of impoverishment | 42.5  (0.6) | 34.7  (1.6) | 42.9  (0.9) | 33.4  (1.5) | 64.8  (0.7) | 52.0  (1.2) |
| **2. Non-spenders (total)** | 47.3  (0.6) | 51.5  (1.7) | 46.5  (1.0) | 51.9  (1.7) | 24.2  (0.7) | 32.4  (1.2) |
| 2a. Non-spender and well | 27.9  (0.5) | 33.1  (1.6) | 28.4  (0.9) | 33.0  (1.7) | 19.2  (0.6) | 25.7  (1.0) |
| 2b. Non-spender with chronic illness in the last 12 months | 15.3  (0.4) | 14.9  (1.2) | 17.1  (0.6) | 17.7  (1.2) | 1.5  (0.1) | 2.4  (0.30 |
| 2c. Non-spender, financial reason, illness in the last 30 days | 0.7  (0.1) | 0.5  (0.2) | 0.1  (0.0) | 0.2  (0.1) | 0.2  (0.0) | 0.6  (0.1) |
| 2c (alt.). Non-spender, financial reason, illness in the last 30 days (alternative definition) | 0.8  (0.1) | 0.6  (0.3) | 0.1  (0.0) | 0.2  (0.1) | 0.5  (0.1) | 0.7  (0.1) |
| 2d. Non-spender, non-financial reasons, illness in the last 30 days | 2.8  (0.2) | 2.3  (0.5) | 0.7  (0.1) | 0.8  (0.2) | 1.9  (0.2) | 2.2  (0.4) |
| 2d (alt.). Non-spender, non-financial reasons, illness in the last 30 days (alternative definition) | 2.7  (0.2) | 2.2  (0.5 | 0.7  (0.1) | 0.8  (0.2) | 1.6  (0.2) | 2.1  (0.4) |
| 2e. Non-spender but sought health care | 0.7  (0.1) | 0.8  (0.3) | 0.3  (0.1) | 0.3  (0.1) | 1.4  (0.1) | 1.4  (0.2) |

Numbers in parentheses are standard errors

^a^ Sum of incidences of risk categories 1a, 1b, 1c, 1d, and 2 = 100%; Sum of incidences of risk categories 1a, 1b, 1c, 1d, 2a, 2b, 2c, 2d, and 2e = 100%; Sum of incidences of risk categories 1a, 1b, 1c, 1d, 2a, 2b, 2c (alt.), 2d (alt.), and 2e = 100%

**Article title:** Financial risk protection in Bangladesh in the era of Universal Health Coverage

**Journal name:** *PLOS ONE*

**S3d Table: Incidences of impoverishment effects and non-spenders (including households forgoing care due to financial reasons) (as % of all households)** **by household head’s education level**

|  |  | | | | | |
| --- | --- | --- | --- | --- | --- | --- |
|  | **2005**  **(n=10,075)** | | **2010**  **(n=12,237)** | | **2016**  **(n=45,976)** | |
| **Risk categories^a^** | no education | secondary and above  education | no education | secondary and above  education | no education | secondary and above  education |
| 1. **Spenders** |  |  |  |  |  |  |
| 1a. Further impoverished | 6.5  (0.6) | 0.2  (0.1) | 6.2  (0.4) | 0.6  (0.2) | 5.9  (0.3) | 0.7  (0.1) |
| 1b. Impoverished | 2.2  (0.4) | 0.2  (0.1) | 2.0  (0.2) | 0.3  (0.1) | 3.5  (0.2) | 0.4  (0.1) |
| 1c. At risk of impoverishment | 8.4  (0.7) | 0.9  (0.2) | 7.1  (0.4) | 1.3  (0.3) | 7.4  (0.3) | 1.7  (0.2) |
| 1d. Not at risk of impoverishment | 36.6  (1.2) | 47.0  (1.4) | 37.8  (1.1) | 43.7  (1.70 | 56.3  (0.8) | 73.5  (1.5) |
| **2. Non-spenders (total)** | 46.3  (1.2) | 51.8  (1.4) | 46.9  (1.3) | 54.1  (1.7) | 27.0  (0.8) | 23.6  (1.4) |
| 2a. Non-spender and well | 26.8  (1.1) | 33.5  (1.3) | 29.0  (1.2) | 32.2  (1.9) | 20.2  (0.7) | 19.6  (1.3) |
| 2b. Non-spender with chronic illness in the last 12 months | 14.9  (0.9) | 15.8  (1.0) | 16.6  (0.7) | 20.8  (1.2) | 2.3  (0.3) | 1.1  (0.2) |
| 2c. Non-spender, financial reason, illness in the last 30 days | 0.9  (0.2) | 0.1  (0.1) | 0.2  (0.1) | 0.1  (0.1) | 0.5  (0.1) | 0.1  (0.0) |
| 2c (alt.). Non-spender, financial reason, illness in the last 30 days (alternative definition) | 0.9  (0.2) | 0.1  (0.1) | 0.2  (0.1) | 0.1  (0.1) | 0.8  (0.1) | 0.3  (0.1) |
| 2d. Non-spender, non-financial reasons, illness in the last 30 days | 3.0  (0.4) | 1.7  (0.4) | 0.8  (0.1) | 0.8  (0.2) | 2.1  (0.3) | 1.9  (0.4) |
| 2d (alt.). Non-spender, non-financial reasons, illness in the last 30 days (alternative definition) | 2.9  (0.4) | 1.7  (0.4) | 0.8  (0.1) | 0.8  (0.2) | 1.8  (0.2) | 1.6  (0.4) |
| 2e. Non-spender but sought health care | 0.8  (0.2) | 0.7  (0.2) | 0.3  (0.1) | 0.2  (0.1) | 1.6  (0.2) | 1.0  (0.2) |

Numbers in parentheses are standard errors

^a^ Sum of incidences of risk categories 1a, 1b, 1c, 1d, and 2 = 100%; Sum of incidences of risk categories 1a, 1b, 1c, 1d, 2a, 2b, 2c, 2d, and 2e = 100%; Sum of incidences of risk categories 1a, 1b, 1c, 1d, 2a, 2b, 2c (alt.), 2d (alt.), and 2e = 100%

**Article title:** Financial risk protection in Bangladesh in the era of Universal Health Coverage

**Journal name:** *PLOS ONE*

**S3e Table:** **Incidence of impoverishment effects and non-spenders (including households forgoing care due to financial reasons) by the presence of chronic illness (%)**

| **Risk categories^a^** |  | | | | | |
| --- | --- | --- | --- | --- | --- | --- |
|  | **2005**  **(n=10,075)** | | **2010**  **(n=12,237)** | | **2016**  **(n=45,976)** | |
|  | **no** | **yes** | **no** | **yes** | **no** | **yes** |
| 1. **Spenders** |  |  |  |  |  |  |
| 1a. Further impoverished | 3.4  (0.3) | 3.9  (0.3) | 4.1  (0.3) | 4.1  (0.3) | 3.2  (0.2) | 4.6  (0.2) |
| 1b. Impoverished | 1.2  (0.2) | 2.0  (0.2) | 1.6  (0.2) | 1.6  (0.2) | 1.3  (0.1) | 3.4  (0.2) |
| 1c. At risk of impoverishment | 4.9  (0.3) | 6.1  (0.4) | 5.0  (0.3) | 6.0  (0.4) | 4.5  (0.2) | 6.6  (0.3) |
| 1d. Not at risk of impoverishment | 35.6  (0.7) | 49.4  (0.8) | 34.3  (1.1) | 50.0  (1.0) | 47.9  (1.0) | 80.1  (0.6) |
| **2. Non-spenders (total)** | 55.0  (0.7) | 38.6  (0.8) | 55.0  (1.2) | 38.3  (1.1) | 43.0  (1.1) | 5.4  (0.4) |
| 2a. Non-spender and well | 51.0  (0.7) | n/a  (n/a) | 54.1  (1.2) | n/a  (n/a) | 38.1  (0.9) | n/a  (n/a) |
| 2b. Non-spender with chronic illness in the last 12 months | n/a  (n/a) | 34.5  (0.8) | n/a  (n/a) | 37.0  (1.0) | n/a  (n/a) | 3.3  (0.3) |
| 2c. Non-spender, financial reason, illness in the last 30 days | 0.4  (0.1) | 1.0  (0.2) | 0.1  (0.0) | 0.2  (0.1) | 0.3  (0.0) | 0.3  (0.0) |
| 2c (alt.). Non-spender, financial reason, illness in the last 30 days (alternative definition) | 0.5  (0.1) | 1.1  (0.2) | 0.1  (0.0) | 0.2  (0.1) | 0.3  (0.0) | 0.7  (0.2) |
| 2d. Non-spender, non-financial reasons, illness in the last 30 days | 2.8  (0.2) | 2.5  (0.2) | 0.6  (0.1) | 0.7  (0.1) | 2.6  (0.3) | 1.2  (0.2) |
| 2d (alt.). Non-spender, non-financial reasons, illness in the last 30 days (alternative definition) | 2.8  (0.2) | 2.4  (0.2) | 0.6  (0.1) | 0.7  (0.1) | 2.6  (0.3) | 0.7  (0.1) |
| 2e. Non-spender but sought health care | 0.8  (0.1) | 0.6  (0.1) | 0.3  (0.1) | 0.3  (0.1) | 2.1  (0.2) | 0.6  (0.1) |

Numbers in parentheses are standard errors

^a^ Sum of incidences of risk categories 1a, 1b, 1c, 1d, and 2 = 100%; Sum of incidences of risk categories 1a, 1b, 1c, 1d, 2a, 2b, 2c, 2d, and 2e = 100%; Sum of incidences of risk categories 1a, 1b, 1c, 1d, 2a, 2b, 2c (alt.), 2d (alt.), and 2e = 100%
