# Supplementary material for: Financial risk protection in health care in Bangladesh in the era of Universal Health Coverage
Source: PLoS One. 2022 Jun 24;17(6):e0269113. doi: 10.1371/journal.pone.0269113 (PMC9231789; doi:10.1371/journal.pone.0269113)
Supplement: S4 Table — a. Incidence of catastrophic health expenditure (%) over time by equity strata; normative food, rent, and utilities method (threshold: OOP≥ 30% of CTP). b. Incidence of catastrophic health expenditure (%) over time by equity strata; normative food, rent, and utilities method (threshold: OOP≥ 20% of CTP). (DOCX) [file pone.0269113.s004.docx]

**Article title:** Financial risk protection in Bangladesh in the era of Universal Health Coverage

**Journal name:** *PLOS ONE*

**S4a Table. Incidence of catastrophic health expenditure (%) over time by equity strata; normative food, rent, and utilities method (30% threshold)**

| **CHE** |  | | |
| --- | --- | --- | --- |
|  | **2005**  **(n=10,075)** | **2010**  **(n=12,237)** | **2016**  **(n=45,976)** |
| **Consumption quintiles** |  |  |  |
| poorest | 32.3 (1.1) | 35.2 (1.4) | 41.4 (0.9) |
| 2nd | 12.5 (0.8) | 12.2 (0.8) | 18.2 (0.6) |
| 3rd | 9.3 (0.7) | 8.4 (0.6) | 14.8 (0.6) |
| 4th | 7.7 (0.7) | 8.4 (0.6) | 15.6 (0.9) |
| richest | 10.0 (0.7) | 10.3 (0.8) | 17.6 (0.8) |
| overall | 14.4 (0.4) | 14.9 (0.5) | 21.5 (0.5) |
| **Area of residence** |  |  |  |
| rural | 16.0 (0.5) | 17.7 (0.6) | 24.5 (0.6) |
| urban | 9.5 (0.6) | 7.2 (0.5) | 13.9 (0.7) |
| **Sex of household head** |  |  |  |
| male | 14.0 (0.4) | 14.1 (0.5) | 21.1 (0.5) |
| female | 18.1 (1.3) | 19.9 (1.1) | 24.3 (0.9) |
| **Level of education of household head** |  |  |  |
| no education | 20.9 (1.0) | 18.3 (0.7) | 25.8 (0.7) |
| below secondary | 11.6 (0.6) | 13.5 (0.7) | 20.3 (0.5) |
| secondary and above | 6.4 (0.7) | 6.3 (0.6) | 12.9 (0.8) |
| **Presence of chronic illness** |  |  |  |
| no | 12.2 (0.5) | 12.3 (0.6) | 11.5 (0.4) |
| yes | 17.1 (0.6) | 17.8 (0.7) | 32.7 (0.6) |

CHE = Catastrophic health expenditure, OOP = Out-of-pocket health expenditure, CTP = Capacity-to-pay

The numbers in parentheses are standard errors; CHE is defined as household OOP expenditure exceeding 30% of household CTP plus any health expenditure by poor households. Therefore, the overall incidence of CHE does not reflect the average of the CHE incidences of the five consumption quintiles

**Article title:** Financial risk protection in Bangladesh in the era of Universal Health Coverage

**Journal name:** *PLOS ONE*

**S4b Table. Incidence of catastrophic health expenditure (%) over time by equity strata; normative food, rent, and utilities method (20% threshold)**

| **CHE** |  | | |
| --- | --- | --- | --- |
|  | **2005**  **(n=10,075)** | **2010**  **(n=12,237)** | **2016**  **(n=45,976)** |
| **Consumption quintiles** |  |  |  |
| poorest | 35.0 (1.1) | 38.6 (1.5) | 46.4 (0.9) |
| 2nd | 18.6 (0.9) | 18.5 (1.0) | 26.9 (0.8) |
| 3rd | 14.7 (0.9) | 14.6 (0.9) | 23.1 (0.7) |
| 4th | 12.3 (0.8) | 13.3 (0.8) | 23.5 (1.0) |
| richest | 13.3 (0.8) | 14.1 (1.0) | 25.1 (1.1) |
| overall | 18.8 (0.4) | 19.8 (0.6) | 29.0 (0.6) |
| **Area of residence** |  |  |  |
| rural | 20.8 (0.5) | 23.2 (0.7) | 32.4 (0.7) |
| urban | 12.8 (0.7) | 10.6 (0.8) | 20.5 (1.0) |
| **Sex of household head** |  |  |  |
| male | 18.5 (0.4) | 19.2 (0.6) | 28.8 (0.6) |
| female | 21.8 (1.4) | 24.0 (1.3) | 30.9 (1.0) |
| **Level of education of household head** |  |  |  |
| no education | 26.4 (1.1) | 23.5 (0.8) | 33.3 (0.8) |
| below secondary | 15.7 (0.7) | 18.9 (0.8) | 28.2 (0.7) |
| secondary and above | 8.9 (0.8) | 9.6 (0.8) | 19.2 (0.9) |
| **Presence of chronic illness** |  |  |  |
| no | 15.7 (0.5) | 16.1 (0.7) | 15.6 (0.6) |
| yes | 22.7 (0.7) | 24.2 (0.8) | 44.0 (0.7) |

CHE = Catastrophic health expenditure, OOP = Out-of-pocket health expenditure, CTP = Capacity-to-pay

Numbers in parentheses are standard errors; CHE is defined as household OOP expenditure exceeding 20% of household CTP plus any health expenditure by poor households. Therefore, the overall incidence of CHE does not reflect the average of the CHE incidences of the five consumption quintiles
