# Supplementary material for: Financial risk protection in health care in Bangladesh in the era of Universal Health Coverage
Source: PLoS One. 2022 Jun 24;17(6):e0269113. doi: 10.1371/journal.pone.0269113 (PMC9231789; doi:10.1371/journal.pone.0269113)
Supplement: S5 Table — (DOCX) [file pone.0269113.s005.docx]

**Article title:** Financial risk protection in Bangladesh in the era of Universal Health Coverage

**Journal name:** *PLOS ONE*

**S5 Table. The levels and distributions of out-of-pocket (OOP) expenditure (alternative calculations)**

|  | **Model 1^a^** | | | | | | **Model 2^b^** | | | | | | |
| --- | --- | --- | --- | --- | --- | --- | --- | --- | --- | --- | --- | --- | --- |
|  | **2005**  **(n=10,075)** | | **2010**  **(n=12,237)** | | **2016**  **(n=45,976)** | | **2005**  **(n=10,075)** | | **2010**  **(n=12,237)** | | **2016**  **(n=45,976)** | | |
|  | | | | | | | | | | | | | |
| **Panel A: Mean annual OOP expenditure (USD)^c^** | | | | | | | | | | | | | |
| Total OOP expenditure | 115.6 | (5.7) | 143.4 | (10.6) | 242.9 | (6.5) | 59.6 | (2.1) | 92.4 | (6.4) | 101.3 | (2.5) |  |
|  |  |  |  |  |  |  |  |  |  |  |  |  |  |
| **Components** | | | | | | | | | | | | | |
| consultation | 10.9 | (0.7) | 10.3 | (0.5) | 15.1 | (1.2) | 7.2 | (0.3) | 10.0 | (0.5) | 11.3 | (0.5) |  |
| medicine | 74.9 | (2.8) | 93.1 | (7.3) | 178.4 | (4.6) | 43.5 | (1.3) | 69.5 | (6.1) | 73.9 | (1.8) |  |
| diagnosis | 13.5 | (1.9) | 17.5 | (1.2) | 35.0 | (1.5) | 1.9 | (0.2) | 6.1 | (0.4) | 8.7 | (0.4) |  |
| hospitalization | 7.2 | (1.2) | 10.8 | (2.4) | 7.6 | (0.6) | 3.6 | (1.1) | 3.9 | (0.5) | 3.7 | (0.3) |  |
| maternity | 3.5 | (0.9) | 3.5 | (1.1) | 1.6 | (0.3) | 2.9 | (0.4) | 2.5 | (0.3) | 1.8 | (0.2) |  |
| contraceptives | n/a | n/a | n/a | n/a | 2.3 | (0.1) | n/a | n/a | n/a | n/a | 1.4 | (0.1) |  |
| dental | n/a | n/a | n/a | n/a | n/a | n/a | 0.2 | (0.0) | 0.3 | (0.1) | 0.3 | (0.0) |  |
| medical assistive products | n/a | n/a | n/a | n/a | n/a | n/a | 0.3 | (0.0) | 0.1 | (0.0) | 0.2 | (0.0) |  |
| immunization | n/a | n/a | n/a | n/a | 3.0 | (1.7) | n/a | n/a | n/a | n/a | n/a | n/a |  |
| tips | 1.3 | (0.8) | 2.5 | (1.9) | 0.6 | (0.1) | n/a | n/a | n/a | n/a | n/a | n/a |  |
| other charges | 4.4 | (0.5) | 5.7 | (0.9) | 0.9 | (0.1) | n/a | n/a | n/a | n/a | n/a | n/a |  |
|  |  |  |  |  |  |  |  |  |  |  |  |  |  |
| **Consumption quintile** | | | | | | | | | | | | | |
| poorest | 50.6 | (7.2) | 65.5 | (7.0) | 108.6 | (6.8) | 17.6 | (0.6) | 27.5 | (0.9) | 29.6 | (0.7) |  |
| 2nd | 70.4 | (6.2) | 85.5 | (5.4) | 161.7 | (6.5) | 29.9 | (0.9) | 44.6 | (1.7) | 55.8 | (1.3) |  |
| 3rd | 91.4 | (7.1) | 121.2 | (8.9) | 210.0 | (8.4) | 42.0 | (1.4) | 64.6 | (2.4) | 80.1 | (2.4) |  |
| 4th | 134.8 | (11.1) | 165.8 | (11.6) | 285.6 | (12.9) | 63.1 | (2.2) | 96.0 | (3.6) | 118.8 | (4.2) |  |
| richest | 231.0 | (22.9) | 279.3 | (44.3) | 448.8 | (19.5) | 145.6 | (9.9) | 229.5 | (29.3) | 222.3 | (7.5) |  |
|  |  |  |  |  |  |  |  |  |  |  |  |  |  |
| **Area of residence** | | | | | | | | | | | | | |
| rural | 107.1 | (6.6) | 157.6 | (14.0) | 242.1 | (7.5) | 50.7 | (1.3) | 84.5 | (3.0) | 92.8 | (2.4) |  |
| urban | 140.7 | (10.8) | 104.9 | (9.5) | 245.1 | (12.8) | 86.0 | (7.3) | 114.1 | (22.4) | 123.1 | (6.7) |  |
|  |  |  |  |  |  |  |  |  |  |  |  |  |  |
| **Sex of household head** | | | | | | | | | | | | | |
| male | 119.1 | (6.2) | 145.5 | (11.9) | 249.2 | (7.0) | 60.5 | (2.2) | 95.5 | (7.3) | 103.1 | (2.7) |  |
| female | 85.8 | (10.3) | 130.8 | (14.2) | 202.5 | (9.9) | 52.1 | (6.3) | 73.5 | (4.8) | 89.4 | (4.0) |  |
|  |  |  |  |  |  |  |  |  |  |  |  |  |  |
| **Education level of household head** | | | | | | | | | | | | | |
| no education | 91.2 | (5.3) | 116.2 | (6.3) | 201.4 | (7.0) | 46.1 | (2.0) | 70.0 | (2.7) | 79.0 | (2.2) |  |
| below secondary | 137.3 | (13.9) | 173.4 | (27.6) | 245.7 | (8.6) | 61.9 | (3.0) | 90.5 | (3.7) | 103.7 | (2.9) |  |
| secondary and above | 159.3 | (16.2) | 175.0 | (23.0) | 351.0 | (19.0) | 102.4 | (9.9) | 168.9 | (35.4) | 156.8 | (7.1) |  |
|  |  |  |  |  |  |  |  |  |  |  |  |  |  |
| **Presence of chronic illness** | | | | | | | | | | | | | |
| no | 83.1 | (7.5) | 88.4 | (5.3) | 92.1 | (5.2) | 36.6 | (1.8) | 53.0 | (2.1) | 47.7 | (1.4) |  |
| yes | 156.7 | (8.6) | 207.2 | (21.4) | 411.0 | (10.9) | 88.7 | (4.2) | 138.2 | (13.4) | 161.1 | (4.1) |  |
|  | | | | | | | | | | | | | |
| **Panel B: OOP expenditure as a percentage of total consumption expenditure** | | | | | | | | | | | | | |
|  | | | | | | | | | | | | | |
| **Consumption quintile** | | | | | | | | | | | | | |
| poorest | 7.1 | (1.0) | 6.7 | (0.7) | 12.4 | (0.8) | 2.5 | (0.1) | 2.8 | (0.1) | 3.4 | (0.1) |  |
| 2nd | 6.4 | (0.6) | 5.7 | (0.4) | 11.5 | (0.5) | 2.7 | (0.1) | 3.0 | (0.1) | 4.0 | (0.1) |  |
| 3rd | 6.1 | (0.5) | 6.1 | (0.4) | 11.2 | (0.4) | 2.8 | (0.1) | 3.2 | (0.1) | 4.3 | (0.1) |  |
| 4th | 6.5 | (0.5) | 6.1 | (0.4) | 11.2 | (0.5) | 3.1 | (0.1) | 3.5 | (0.1) | 4.6 | (0.1) |  |
| richest | 5.2 | (0.5) | 5.0 | (0.8) | 8.9 | (0.4) | 3.3 | (0.2) | 4.1 | (0.5) | 4.4 | (0.1) |  |
| overall | 5.9 | (0.3) | 5.6 | (0.4) | 10.3 | (0.3) | 3.0 | (0.1) | 3.6 | (0.2) | 4.3 | (0.1) |  |
|  |  |  |  |  |  |  |  |  |  |  |  |  |  |
| **Area of residence** |  |  |  |  |  |  |  |  |  |  |  |  |  |
| rural | 6.3 | (0.4) | 7.1 | (0.6) | 11.4 | (0.3) | 3.0 | (0.1) | 3.8 | (0.1) | 4.4 | (0.1) |  |
| urban | 5.1 | (0.4) | 3.0 | (0.3) | 8.2 | (0.4) | 3.1 | (0.3) | 3.3 | (0.6) | 4.1 | (0.2) |  |
|  |  |  |  |  |  |  |  |  |  |  |  |  |  |
| **Sex of household head** | | | | | | | | | | | | | |
| male | 5.9 | (0.3) | 5.5 | (0.4) | 10.3 | (0.3) | 3.0 | (0.1) | 3.6 | (0.2) | 4.3 | (0.1) |  |
| female | 5.2 | (0.6) | 6.4 | (0.7) | 10.0 | (0.5) | 3.1 | (0.3) | 3.6 | (0.2) | 4.4 | (0.2) |  |
|  |  |  |  |  |  |  |  |  |  |  |  |  |  |
| **Education level of household head** | | | | | | | | | | | | | |
| no education | 6.2 | (0.4) | 5.9 | (0.3) | 10.6 | (0.4) | 3.1 | (0.1) | 3.6 | (0.1) | 4.2 | (0.1) |  |
| below secondary | 6.5 | (0.7) | 6.7 | (1.1) | 10.4 | (0.4) | 3.0 | (0.1) | 3.5 | (0.1) | 4.4 | (0.1) |  |
| secondary and above | 4.6 | (0.5) | 4.0 | (0.6) | 9.6 | (0.6) | 2.9 | (0.3) | 3.8 | (0.7) | 4.3 | (0.2) |  |
|  |  |  |  |  |  |  |  |  |  |  |  |  |  |
| **Presence of chronic illness** | | | | | | | | | | | | | |
| no | 4.6 | (0.4) | 3.8 | (0.2) | 4.3 | (0.2) | 2.0 | (0.1) | 2.3 | (0.1) | 2.2 | (0.1) |  |
| yes | 7.1 | (0.4) | 7.3 | (0.8) | 15.7 | (0.4) | 4.0 | (0.2) | 4.9 | (0.4) | 6.2 | (0.1) |  |
|  |  |  |  |  |  |  |  |  |  |  |  |  |  |
| **Panel C: OOP expenditure as a percentage of household capacity-to-pay (CTP)** | | | | | | | | | | | | | |
|  | | | | | | | | | | | | | |
| **consumption quintiles** | | | | | | | | | | | | | |
| poorest ^d^ | -464.0 | (139.6) | -713.4 | (398.2) | 203.6 | (18.9) | -161.6 | (43.8) | -299.5 | (164.9) | 55.5 | (3.6) |  |
| 2nd | 22.8 | (2.0) | 19.5 | (1.2) | 31.2 | (1.2) | 9.7 | (0.3) | 10.2 | (0.4) | 10.8 | (0.2) |  |
| 3rd | 13.8 | (1.1) | 13.5 | (1.0) | 21.7 | (0.9) | 6.3 | (0.2) | 7.2 | (0.3) | 8.3 | (0.2) |  |
| 4th | 11.1 | (0.9) | 10.4 | (0.7) | 17.5 | (0.8) | 5.2 | (0.2) | 6.0 | (0.2) | 7.3 | (0.2) |  |
| richest | 6.4 | (0.6) | 6.3 | (1.0) | 10.9 | (0.5) | 4.0 | (0.3) | 5.2 | (0.6) | 5.4 | (0.2) |  |
| overall | 10.0 | (0.5) | 9.8 | (0.8) | 16.6 | (0.5) | 5.2 | (0.2) | 6.3 | (0.4) | 6.9 | (0.2) |  |
|  |  |  |  |  |  |  |  |  |  |  |  |  |  |
| **Area of residence** |  |  |  |  |  |  |  |  |  |  |  |  |  |
| rural | 12.0 | (0.8) | 13.9 | (1.2) | 19.6 | (0.6) | 5.7 | (0.2) | 7.5 | (0.2) | 7.5 | (0.2) |  |
| urban | 7.3 | (0.6) | 4.4 | (0.5) | 12.0 | (0.7) | 4.5 | (0.4) | 4.8 | (0.8) | 6.0 | (0.2) |  |
|  |  |  |  |  |  |  |  |  |  |  |  |  |  |
| **Sex of household head** | | | | | | | | | | | | | |
| male | 10.2 | (0.5) | 9.6 | (0.8) | 16.8 | (0.5) | 5.2 | (0.2) | 6.3 | (0.4) | 6.9 | (0.2) |  |
| female | 8.6 | (1.1) | 11.6 | (1.4) | 15.9 | (0.8) | 5.2 | (0.5) | 6.5 | (0.4) | 7.0 | (0.3) |  |
|  |  |  |  |  |  |  |  |  |  |  |  |  |  |
| **Education level of household head** | | | | | | | | | | | | | |
| no education | 13.7 | (0.8) | 13.0 | (0.7) | 20.1 | (0.8) | 6.9 | (0.3) | 7.8 | (0.3) | 7.9 | (0.2) |  |
| below secondary | 10.8 | (1.1) | 11.6 | (1.8) | 16.9 | (0.6) | 4.9 | (0.2) | 6.1 | (0.2) | 7.1 | (0.2) |  |
| secondary and above | 6.0 | (0.6) | 5.4 | (0.8) | 12.8 | (0.8) | 3.9 | (0.4) | 5.2 | (0.9) | 5.7 | (0.2) |  |
|  | | | | | | | | | | | | | |
| **Presence of chronic illness** | | | | | | | | | | | | | |
| no | 8.4 | (0.8) | 7.0 | (0.5) | 7.4 | (0.5) | 3.7 | (0.2) | 4.2 | (0.2) | 3.8 | (0.1) |  |
| yes | 11.5 | (0.7) | 12.1 | (1.3) | 24.1 | (0.8) | 6.5 | (0.3) | 8.1 | (0.6) | 9.5 | (0.2) |  |

n/a = not available; numbers in parentheses are standard errors

^a^ Model 1: out-of-pocket (OOP) expenditure comes from HIES’s health module when used as a separate variable, but the OOP component of total consumption expenditure (thus, of capacity-to-pay) is sourced from the HIES consumption module

^b^ Model 2: OOP expenses data comes from HIES’s health module, both as a separate variable and as a component of total consumption expenditure

^c^ BDT to USD conversion rates: USD 1 = BDT 78.468 (source: https://data.worldbank.org/indicator/PA.NUS.FCRF?locations=BD)

^d^ CTP = consumption expenditure – subsistence expenditure. Negative values of OOP as a percentage of CTP for the poorest quintile mean that an average household in the bottom quintile had total consumption expenditure less than their respective subsistence expenditure.
