# Supplementary material for: Financial risk protection in health care in Bangladesh in the era of Universal Health Coverage
Source: PLoS One. 2022 Jun 24;17(6):e0269113. doi: 10.1371/journal.pone.0269113 (PMC9231789; doi:10.1371/journal.pone.0269113)
Supplement: S6 Table — (DOCX) [file pone.0269113.s006.docx]

**Article title:** Financial risk protection in Bangladesh in the era of Universal Health Coverage

**Journal name:** *PLOS ONE*

**S6 Table. Incidences of impoverishment effects and non-spenders (alternative calculations)**

| **Risk categories** ^c^ | **Model 1^a^** | | | **Model 2^b^** | | |
| --- | --- | --- | --- | --- | --- | --- |
|  | **2005**  **(n=10,075)** | **2010**  **(n=12,237)** | **2016**  **(n=45,976)** | **2005**  **(n=10,075)** | **2010**  **(n=12,237)** | **2016**  **(n=45,976)** |
| 1. **Spenders** |  |  |  |  |  |  |
| 1a. Further impoverished | 4.3 (0.2) | 4.7 (0.3) | 4.5 (0.2) | 8.9 (0.3) | 8.7 (0.4) | 5.7 (0.2) |
| 1b. Impoverished | 4.0 (0.2) | 3.6 (0.2) | 5.8 (0.2) | 1.2 (0.1) | 1.3 (0.1) | 1.3 (0.1) |
| 1c. At risk of impoverishment | 5.7 (0.2) | 5.5 (0.3) | 5.8 (0.2) | 9.6 (0.3) | 9.0 (0.4) | 6.4 (0.2) |
| 1d. Not at risk of impoverishment | 38.2 (0.5) | 38.9 (0.9) | 58.5 (0.7) | 73.8 (0.5) | 70.9 (0.8) | 77.7 (0.6) |
| **2. Non-spenders** ^c^ **(total)** | 47.8 (0.5) | 47.3 (1.0) | 25.3 (0.7) | 6.5 (0.3) | 10.1 (0.7) | 8.9 (0.4) |
|  |  |  |  |  |  |  |
| 2a. Non-spender and well | 28.4 (0.5) | 29.0 (0.9) | 20.1 (0.6) | 3.8 (0.2) | 5.7 (0.5) | 5.7 (0.3) |
| 2b. Non-spender with chronic illness in the last 12 months | 15.3 (0.4) | 17.1 (0.5) | 1.6 (0.1) | 0.5 (0.1) | 1.1 (0.2) | 1.0 (0.1) |
| 2c. Non-spender, financial reason, illness in the last 30 days | 0.7 (0.1) | 0.1 (0.0) | 0.3 (0.0) | 0.1 (0.0) | 0.1 (0.0) | 0.1 (0.0) |
| 2c (alt.). Non-spender, financial reason, illness in the last 30 days (alternative definition) | 0.8 (0.1) | 0.1 (0.0) | 0.5 (0.1) | 0.1 (0.0) | 0.1 (0.0) | 0.1 (0.0) |
| 2d. Non-spender, non-financial reasons, illness in the last 30 days | 2.7 (0.2) | 0.7 (0.1) | 2.0 (0.2) | 0.4 (0.1) | 0.3 (0.1) | 0.4 (0.1) |
| 2d (alt.). Non-spender, non-financial reasons, illness in the last 30 days (alternative definition) | 2.6 (0.2) | 0.7 (0.1) | 1.7 (0.2) | 0.4 (0.1) | 0.3 (0.1) | 0.4 (0.1) |
| 2e. Non-spender but sought health care | 0.7 (0.1) | 0.3 (0.1) | 1.4 (0.1) | 1.6 (0.1) | 2.9 (0.3) | 1.7 (0.1) |

Households are at risk of impoverishment if consumption after OOP expenditure is between 100% and 120% of subsistence expenditure; numbers in parentheses are standard errors

^a^ Model 1: out-of-pocket (OOP) expenditure comes from HIES’s health module when used as a separate variable, but the OOP component of total consumption expenditure (thus, of capacity-to-pay) is sourced from the HIES consumption module

^b^ Model 2: OOP expenses data comes from HIES’s health module, both as a separate variable and as a component of total consumption expenditure

^c^ Sum of incidences of risk categories 1a, 1b, 1c, 1d, and 2 = 100%; Sum of incidences of risk categories 1a, 1b, 1c, 1d, 2a, 2b, 2c, 2d, and 2e = 100%; Sum of incidences of risk categories 1a, 1b, 1c, 1d, 2a, 2b, 2c (alt.), 2d (alt.), and 2e = 100%
