# Supplementary material for: Financial risk protection in health care in Bangladesh in the era of Universal Health Coverage
Source: PLoS One. 2022 Jun 24;17(6):e0269113. doi: 10.1371/journal.pone.0269113 (PMC9231789; doi:10.1371/journal.pone.0269113)
Supplement: S7 Table — (DOCX) [file pone.0269113.s007.docx]

**Article title:** Financial risk protection in Bangladesh in the era of Universal Health Coverage

**Journal name:** *PLOS ONE*

**S7 Table. The levels and distributions of the incidence of catastrophic health expenditure (%); normative food, housing, and utilities method, 40% threshold (alternative calculations)**

|  | **Model 1^a^** | | | **Model 2^b^** | | |
| --- | --- | --- | --- | --- | --- | --- |
|  | **2005**  **(n=10,075)** | **2010**  **(n=12,237)** | **2016**  **(n=45,976)** | **2005**  **(n=10,075)** | **2010**  **(n=12,237)** | **2016**  **(n=45,976)** |
| **Consumption quintiles** |  |  |  |  |  |  |
| poorest | 37.1 (1.1) | 39.1 (1.4) | 45.8 (0.8) | 58.6 (1.2) | 59.9 (1.3) | 43.6 (0.9) |
| 2nd | 13.3 (0.8) | 12.2 (0.8) | 19.0 (0.6) | 3.8 (0.5) | 4.6 (0.5) | 5.3 (0.3) |
| 3rd | 8.2 (0.7) | 7.6 (0.6) | 13.4 (0.6) | 1.4 (0.3) | 1.3 (0.3) | 2.4 (0.2) |
| 4th | 5.7 (0.6) | 6.7 (0.6) | 11.1 (0.6) | 0.9 (0.2) | 1.2 (0.2) | 1.6 (0.2) |
| richest | 3.9 (0.5) | 3.5 (0.4) | 6.8 (0.4) | 0.8 (0.2) | 1.1 (0.2) | 1.5 (0.2) |
| Overall ^c^ | 13.6 (0.4) | 13.8 (0.5) | 19.2 (0.4) | 13.1 (0.3) | 13.6 (0.5) | 10.9 (0.4) |
| **Area of residence** |  |  |  |  |  |  |
| rural | 15.1 (0.4) | 16.4 (0.6) | 22.0 (0.5) | 15.5 (0.4) | 16.7 (0.7) | 13.0 (0.4) |
| urban | 9.2 (0.6) | 6.7 (0.5) | 12.1 (0.7 | 6.2 (0.4) | 5.3 (0.4) | 5.5 (0.4) |
| **Sex of household head** |  |  |  |  |  |  |
| male | 13.2 (0.4) | 13.0 (0.5) | 18.7 (0.4) | 11.7 (0.3) | 12.1 (0.5) | 9.9 (0.3) |
| female | 17.9 (1.3) | 18.9 (1.1) | 22.5 (0.9) | 25.5 (1.4) | 23.2 (1.3) | 17.2 (0.8) |
| **Level of education of household head** |  |  |  |  |  |  |
| no education | 17.3 (0.5) | 16.9 (0.7) | 23.2 (0.6) | 19.4 (0.6) | 19.5 (0.8) | 15.8 (0.6) |
| below secondary | 10.8 (0.6) | 12.6 (0.7) | 18.0 (0.5) | 7.5 (0.5) | 9.3 (0.6) | 8.8 (0.3) |
| secondary and above | 6.2 (0.7) | 6.0 (0.6) | 11.5 (0.7) | 1.9 (0.4) | 2.9 (0.4) | 3.2 (0.3) |
| **Presence of chronic illness** |  |  |  |  |  |  |
| no | 11.8 (0.5) | 11.7 (0.5) | 10.5 (0.4) | 12.2 (0.4) | 13.2 (0.7) | 8.7 (0.4) |
| yes | 16.0 (0.6) | 16.2 (0.7) | 28.9 (0.6) | 14.2 (0.5) | 14.1 (0.6) | 13.3 (0.4) |

Numbers in parentheses are standard errors

^a^ Model 1: out-of-pocket (OOP) expenditure comes from HIES’s health module when used as a separate variable, but the OOP component of total consumption expenditure (thus, of capacity-to-pay) is sourced from the HIES consumption module

^b^ Model 2: OOP expenses data comes from HIES’s health module, both as a separate variable and as a component of total consumption expenditure

^c^ Catastrophic health expenditure (CHE) is defined as household OOP expenditure exceeding 40% of household capacity-to-pay plus any health expenditure by poor households. Therefore, the overall incidence of CHE does not reflect the average of the CHE incidences of the five consumption quintiles
