# Supplementary material for: Financial risk protection in health care in Bangladesh in the era of Universal Health Coverage
Source: PLoS One. 2022 Jun 24;17(6):e0269113. doi: 10.1371/journal.pone.0269113 (PMC9231789; doi:10.1371/journal.pone.0269113)
Supplement: S8 Table — a. The levels and distributions of the incidence of catastrophic health expenditure (%) by year; budget share method, 10% threshold (alternative calculations). b. The levels and distributions of the incidence of catastrophic health expenditure (%) by year; budget share method, 25% threshold (alternative calculations). c. The levels and distributions of the incidence of catastrophic health expenditure (%) by year; actual food expenditure method, 40% threshold (alternative calculations). d. The levels and distributions of the incidence of catastrophic health expenditure (%) by year; normative food expenditure method, 40% threshold (alternative calculations). (DOCX) [file pone.0269113.s008.docx]

**Article title:** Financial risk protection in Bangladesh in the era of Universal Health Coverage

**Journal name:** *PLOS ONE*

**S8a Table. The levels and distributions of the incidence of catastrophic health expenditure (%) by year; budget share method, 10% threshold (alternative calculations)**

|  | **Model 1^a^** | | | **Model 2^b^** | | |
| --- | --- | --- | --- | --- | --- | --- |
|  | **2005**  **(n=10,075)** | **2010**  **(n=12,237)** | **2016**  **(n=45,976)** | **2005**  **(n=10,075)** | **2010**  **(n=12,237)** | **2016**  **(n=45,976)** |
| **Consumption quintiles** |  |  |  |  |  |  |
| poorest | 14.4 (0.8) | 14.4 (0.2) | 25.6 (0.7) | 3.2 (0.4) | 4.3 (0.4) | 8.5 (0.4) |
| 2nd | 13.4 (0.8) | 13.2 (0.1) | 25.9 (0.8) | 4.1 (0.5) | 5.4 (0.5) | 9.7 (0.4) |
| 3rd | 13.8 (0.8) | 14.5 (0.2) | 25.9 (0.8) | 4.3 (0.5) | 6.2 (0.6) | 10.7 (0.5) |
| 4th | 13.6 (0.8) | 15.0 (0.2) | 26.1 (1.0) | 5.1 (0.5) | 7.3 (0.6) | 11.6 (0.7) |
| richest | 11.6 (0.8) | 10.1 (0.1) | 23.3 (1.0) | 6.5 (0.6) | 8.7 (0.7) | 11.5 (0.7) |
| overall | 13.4 (0.4) | 13.4 (0.5) | 25.4 (0.5) | 4.7 (0.2) | 6.4 (0.3) | 10.4 (0.3) |
| **Area of residence** |  |  |  |  |  |  |
| rural | 13.7 (0.4) | 15.2 (0.6) | 27.0 (0.6) | 4.7 (0.3) | 7.1 (0.4) | 10.8 (0.4) |
| urban | 12.4 (0.7) | 8.6 (0.7) | 21.2 (1.2) | 4.5 (0.5) | 4.5 (0.5) | 9.4 (0.6) |
| **Sex of household head** |  |  |  |  |  |  |
| male | 13.3 (0.4) | 13.0 (0.5) | 25.2 (0.6) | 4.5 (0.2) | 6.3 (0.3) | 10.2 (0.3) |
| female | 14.2 (1.2) | 16.0 (1.1) | 26.6 (0.9) | 6.0 (0.9) | 6.9 (0.7) | 11.9 (0.6) |
| **Level of education of household head** |  |  |  |  |  |  |
| no education | 13.6 (0.5) | 13.7 (0.6) | 25.6 (0.6) | 4.7 (0.3) | 6.5 (0.4) | 10.3 (0.4) |
| below secondary | 14.2 (0.7) | 14.8 (0.7) | 25.7 (0.6) | 4.7 (0.4) | 6.3 (0.4) | 10.6 (0.4) |
| secondary and above | 11.1 (0.9) | 9.8 (0.8) | 23.8 (1.1) | 4.4 (0.6) | 6.2 (0.7) | 9.9 (0.6) |
| **Presence of chronic illness** |  |  |  |  |  |  |
| no | 10.2 (0.4) | 9.5 (0.5) | 11.1 (0.5) | 1.9 (0.2) | 3.0 (0.2) | 3.6 (0.2) |
| yes | 17.4 (0.6) | 18.0 (0.7) | 41.3 (0.7) | 8.0 (0.4) | 10.3 (0.5) | 18.0 (0.5) |

Numbers in parentheses are standard errors

^a^ Model 1: out-of-pocket (OOP) expenditure comes from HIES’s health module when used as a separate variable, but the OOP component of total consumption expenditure (thus, of capacity-to-pay) is sourced from the HIES consumption module

^b^ Model 2: OOP expenses data comes from HIES’s health module, both as a separate variable and as a component of total consumption expenditure

**Article title:** Financial risk protection in Bangladesh in the era of Universal Health Coverage

**Journal name:** *PLOS ONE*

**S8b Table. The levels and distributions of the incidence of catastrophic health expenditure (%) by year; budget share method, 25% threshold (alternative calculations)**

|  | **Model 1^a^** | | | **Model 2^b^** | | |
| --- | --- | --- | --- | --- | --- | --- |
|  | **2005**  **(n=10,075)** | **2010**  **(n=12,237)** | **2016**  **(n=45,976)** | **2005**  **(n=10,075)** | **2010**  **(n=12,237)** | **2016**  **(n=45,976)** |
| **Consumption quintiles** |  |  |  |  |  |  |
| poorest | 5.1 (0.5) | 5.4 (0.5) | 11.8 (0.5) | 0.4 (0.2) | 0.3 (0.1) | 1.1 (0.1) |
| 2nd | 5.1 (0.5) | 4.9 (0.5) | 11.1 (0.5) | 0.4 (0.2) | 0.5 (0.2) | 1.3 (0.1) |
| 3rd | 5.5 (0.5) | 5.2 (0.5) | 11.1 (0.5) | 0.6 (0.2) | 0.5 (0.1) | 1.5 (0.2) |
| 4th | 5.4 (0.5) | 6.1 (0.6) | 11.3 (0.6) | 0.8 (0.2) | 1.0 (0.2) | 1.9 (0.2) |
| richest | 4.4 (0.5) | 3.9 (0.4) | 8.9 (0.5) | 1.2 (0.3) | 1.8 (0.3) | 2.3 (0.2) |
| overall | 5.1 (0.2) | 5.1 (0.3) | 10.9 (0.3) | 0.7 (0.1) | 0.8 (0.1) | 1.6 (0.1) |
| **Area of residence** |  |  |  |  |  |  |
| rural | 5.1 (0.3) | 5.8 (0.3) | 11.8 (0.3) | 0.7 (0.1) | 0.9 (0.1) | 1.7 (0.1) |
| urban | 5.0 (0.5) | 3.2 (0.4) | 8.4 (0.5) | 0.7 (0.2) | 0.6 (0.1) | 1.3 (0.2) |
| **Sex of household head** |  |  |  |  |  |  |
| male | 5.1 (0.3) | 4.8 (0.3) | 10.7 (0.3) | 0.7 (0.1) | 0.8 (0.1) | 1.6 (0.1) |
| female | 5.1 (0.8) | 6.7 (0.7) | 11.8 (0.6) | 0.8 (0.3) | 1.1 (0.2) | 2.1 (0.2) |
| **Level of education of household head** |  |  |  |  |  |  |
| no education | 5.2 (0.3) | 5.2 (0.3) | 11.3 (0.4) | 0.6 (0.1) | 0.8 (0.1) | 1.7 (0.1) |
| below secondary | 5.4 (0.4) | 5.4 (0.4) | 10.7 (0.4) | 0.8 (0.2) | 0.9 (0.2) | 1.5 (0.1) |
| secondary and above | 4.2 (0.5) | 4.2 (0.5) | 10.0 (0.7) | 0.6 (0.2) | 0.7 (0.2) | 1.6 (0.2) |
| **Presence of chronic illness** |  |  |  |  |  |  |
| no | 3.7 (0.3) | 3.6 (0.3) | 4.2 (0.3) | 0.2 (0.1) | 0.3 (0.1) | 0.4 (0.1) |
| yes | 6.8 (0.4) | 6.8 (0.4) | 18.3 (0.5) | 1.3 (0.2) | 1.4 (0.2) | 3.0 (0.2) |

Numbers in parentheses are standard errors

^a^ Model 1: out-of-pocket (OOP) expenditure comes from HIES’s health module when used as a separate variable, but the OOP component of total consumption expenditure (thus, of capacity-to-pay) is sourced from the HIES consumption module

^b^ Model 2: OOP expenses data comes from HIES’s health module, both as a separate variable and as a component of total consumption expenditure

**Article title:** Financial risk protection in Bangladesh in the era of Universal Health Coverage

**Journal name:** *PLOS ONE*

**S8c Table. The levels and distributions of the incidence of catastrophic health expenditure (%) by year; actual food expenditure method, 40% threshold (alternative calculations)**

|  | **Model 1^a^** | | | **Model 2^b^** | | |
| --- | --- | --- | --- | --- | --- | --- |
|  | **2005**  **(n=10,075)** | **2010**  **(n=12,237)** | **2016**  **(n=45,976)** | **2005**  **(n=10,075)** | **2010**  **(n=12,237)** | **2016**  **(n=45,976)** |
| **Consumption quintiles** |  |  |  |  |  |  |
| poorest | 13.2 (0.8) | 12.8 (0.9) | 20.0 (0.6) | 2.0 (0.3) | 1.6 (0.3) | 4.0 (0.4) |
| 2nd | 12.0 (0.8) | 10.5 (0.7) | 17.7 (0.6) | 2.0 (0.3) | 1.9 (0.3) | 3.0 (0.2) |
| 3rd | 11.5 (0.8) | 10.5 (0.8) | 15.9 (0.6) | 1.8 (0.3) | 1.7 (0.3) | 2.8 (0.3) |
| 4th | 9.0 (0.7) | 9.6 (0.7) | 15.0 (0.7) | 1.6 (0.3) | 2.2 (0.3) | 2.7 (0.3) |
| richest | 6.5 (0.6) | 5.8 (0.5) | 10.2 (0.6) | 1.5 (0.3) | 2.5 (0.3) | 2.5 (0.2) |
| overall | 10.4 (0.3) | 9.9 (0.4) | 15.7 (0.4) | 1.8 (0.1) | 2.0 (0.2) | 3.0 (0.1) |
| **Area of residence** |  |  |  |  |  |  |
| rural | 11.0 (0.4) | 11.3 (0.5) | 17.5 (0.4) | 2.0 (0.2) | 2.2 (0.2) | 3.3 (0.2) |
| urban | 8.8 (0.6) | 5.9 (0.5) | 11.2 (0.6) | 1.2 (0.2) | 1.2 (0.2) | 2.2 (0.2) |
| **Sex of household head** |  |  |  |  |  |  |
| male | 10.5 (0.3) | 9.6 (0.4) | 15.6 (0.4) | 1.7 (0.1) | 2.0 (0.2) | 2.9 (0.1) |
| female | 10.1 (1.0) | 11.5 (0.9) | 16.8 (0.7) | 2.4 (0.5) | 1.9 (0.3) | 3.7 (0.3) |
| **Level of education of household head** |  |  |  |  |  |  |
| no education | 11.4 (0.5) | 10.9 (0.5) | 17.6 (0.5) | 2.0 (0.2) | 2.3 (0.2) | 3.6 (0.2) |
| below secondary | 10.6 (0.6) | 10.2 (0.6) | 15.3 (0.5) | 1.8 (0.3) | 1.8 (0.2) | 2.7 (0.2) |
| secondary and above | 6.7 (0.7) | 5.7 (0.6) | 12.1 (0.8) | 1.0 (0.3) | 1.2 (0.3) | 2.2 (0.3) |
| **Presence of chronic illness** |  |  |  |  |  |  |
| no | 8.3 (0.4) | 7.3 (0.4) | 7.0 (0.3) | 0.8 (0.1) | 0.9 (0.1) | 1.0 (0.1) |
| yes | 13.1 (0.5) | 12.8 (0.6) | 25.5 (0.5) | 2.9 (0.3) | 3.2 (0.3) | 5.2 (0.2) |

Numbers in parentheses are standard errors

^a^ Model 1: out-of-pocket (OOP) expenditure comes from HIES’s health module when used as a separate variable, but the OOP component of total consumption expenditure (thus, of capacity-to-pay) is sourced from the HIES consumption module

^b^ Model 2: OOP expenses data comes from HIES’s health module, both as a separate variable and as a component of total consumption expenditure

**S8d Table. The levels and distributions of the incidence of catastrophic health expenditure (%) by year; normative food expenditure method, 40% threshold (alternative calculations)**

|  | **Model 1^a^** | | | **Model 2^b^** | | |
| --- | --- | --- | --- | --- | --- | --- |
|  | **2005**  **(n=10,075)** | **2010**  **(n=12,237)** | **2016**  **(n=45,976)** | **2005**  **(n=10,075)** | **2010**  **(n=12,237)** | **2016**  **(n=45,976)** |
| **Consumption quintiles** |  |  |  |  |  |  |
| poorest | 15.4 (0.9) | 15.5 (0.9) | 23.1 (0.6) | 14.0 (0.8) | 16.6 (0.9) | 15.1 (0.5) |
| 2nd | 13.3 (0.8) | 12.2 (0.8) | 19.0 (0.6) | 3.8 (0.5) | 4.6 (0.5) | 5.3 (0.3) |
| 3rd | 8.2 (0.7) | 7.6 (0.6) | 13.4 (0.6) | 1.4 (0.3) | 1.3 (0.3) | 2.4 (0.2) |
| 4th | 5.7 (0.6) | 6.7 (0.6) | 11.1 (0.6) | 0.9 (0.2) | 1.2 (0.2) | 1.6 (0.2) |
| richest | 3.9 (0.5) | 3.5 (0.4) | 6.8 (0.4) | 0.8 (0.2) | 1.1 (0.2) | 1.5 (0.2) |
| overall | 9.3 (0.3) | 9.1 (0.4) | 14.7 (0.3) | 4.2 (0.2) | 5.0 (0.3) | 5.2 (0.2) |
| **Area of residence** |  |  |  |  |  |  |
| rural | 9.9 (0.4) | 10.5 (0.5) | 16.4 (0.4) | 4.8 (0.3) | 5.9 (0.3) | 6.0 (0.2) |
| urban | 7.5 (0.6) | 5.3 (0.5) | 10.1 (0.6) | 2.4 (0.3) | 2.4 (0.2) | 2.9 (0.3) |
| **Sex of household head** |  |  |  |  |  |  |
| male | 9.2 (0.3) | 8.9 (0.4) | 14.8 (0.4) | 4.0 (0.2) | 4.8 (0.3) | 5.1 (0.2) |
| female | 9.7 (1.0) | 10.3 (0.8) | 14.0 (0.7) | 5.5 (0.8) | 5.8 (0.6) | 5.8 (0.4) |
| **Level of education of household head** |  |  |  |  |  |  |
| no education | 10.7 (0.5) | 10.0 (0.5) | 16.0 (0.5) | 5.9 (0.3) | 6.2 (0.4) | 6.7 (0.3) |
| below secondary | 8.5 (0.6) | 9.6 (0.6) | 14.7 (0.5) | 2.6 (0.3) | 4.2 (0.4) | 4.8 (0.2) |
| secondary and above | 5.9 (0.7) | 5.3 (0.6) | 10.7 (0.7) | 1.2 (0.3) | 2.1 (0.4) | 2.1 (0.2) |
| **Presence of chronic illness** |  |  |  |  |  |  |
| no | 7.7 (0.4) | 7.0 (0.4) | 6.9 (0.3) | 2.8 (0.2) | 3.8 (0.3) | 2.8 (0.2) |
| yes | 11.3 (0.5) | 11.5 (0.6) | 23.3 (0.5) | 5.8 (0.4) | 6.3 (0.4) | 7.8 (0.3) |

Numbers in parentheses are standard errors

^a^ Model 1: out-of-pocket (OOP) expenditure comes from HIES’s health module when used as a separate variable, but the OOP component of total consumption expenditure (thus, of capacity-to-pay) is sourced from the HIES consumption module

^b^ Model 2: OOP expenses data comes from HIES’s health module, both as a separate variable and as a component of total consumption expenditure
